# A Biomarker‐Driven Ovary–Endometrium Organ‐on‐a‐Chip Mimicking 3D Multicellular Complexity and Menstrual Cyclicity for Predicting Reproductive Toxicity

**DOI:** 10.1002/advs.202511098

**Published:** 2026-01-15

**Authors:** Soo‐Rim Kim, Eun‐kyung Min, Choon‐Mi Lee, Chan Hum Park, Byung‐Chul Oh, YunJae Jung, In‐Sun Hong

**Affiliations:** ^1^ Department of Health Sciences and Technology GAIHST Gachon University Incheon 21999 Republic of Korea; ^2^ Department of Molecular Medicine School of Medicine Gachon University Incheon 406–840 Republic of Korea; ^3^ Department of Microbiology College of Medicine Gachon University Incheon 21999 Republic of Korea; ^4^ Department of Otolaryngology‐Head and Neck Surgery Chuncheon Sacred Heart Hospital Hallym University College of Medicine Chuncheon 24252 Republic of Korea; ^5^ Department of Physiology Lee Gil Ya Cancer and Diabetes Institute Gachon University College of Medicine Incheon 21999 Republic of Korea

**Keywords:** ANGPTL4, female reproductive system, organ‐on‐a‐chip, reproductive toxicity, SERPINB2

## Abstract

Reliable prediction of reproductive toxicity remains a critical challenge in drug development and environmental safety. Here, a biomarker‐integrated, fluorescent reporter‐based reproductive organ‐on‐a‐chip platform that recapitulates the multicellular composition, 3D architecture, endocrine signaling, and cyclic dynamics of the human menstrual cycle, is presented. The system is constructed using primary human theca, granulosa, endometrial stromal and stem cells, vascular endothelial cells, uterine macrophages, and myometrial smooth muscle cells, compartmentalized within collagen–hyaluronic acid hydrogels. Early‐response toxicity biomarkers—ANGPTL4 (ovary) and SERPINB2 (endometrium)—are genetically linked to mCherry or GFP fluorescent reporters, enabling real‐time, cell‐type‐specific visualization of toxicant‐induced stress. Transcriptomic profiling, KEGG pathway enrichment, and gene knockdown studies confirm ANGPTL4 and SERPINB2 as functional mediators of toxic injury, not just passive indicators. Upon exposure to dioxin and other reproductive toxicants, the platform shows strong, region‐specific fluorescent responses that preceded changes detected by conventional cytotoxicity assays. This system demonstrates high sensitivity, temporal precision, and mechanistic insight, offering a scalable and physiologically relevant tool for high‐content reproductive toxicology screening. Furthermore, it supports endocrine crosstalk between the ovary and uterus, and dynamic responses across the menstrual cycle, enabling future applications in personalized toxicity prediction and preclinical safety evaluation.

## Graphical abstract

We present a dual‐organ, biomarker‐integrated ovaryendometrium organ‐on‐a‐chip that recapitulates 3D tissue complexity, menstrual cycle dynamics, and hormonal crosstalk. This platform enables real‐time, cell‐typespecific fluorescent readouts of reproductive toxicity using ANGPTL4 and SERPINB2 as early‐response reporters. The system demonstrates superior temporal sensitivity and mechanistic insight compared to conventional cytotoxicity assays. It offers a scalable, physiologically relevant tool for personalized toxicity prediction and high‐content reproductive safety screening.

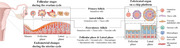

## 1 Introduction

Successful human reproduction relies heavily on the dynamic, hormonally regulated interaction between the ovary and the endometrium, which is the specialized epithelial lining of the uterus.^[^ ^1^, ^2^ ^]^ The endometrium is essential for embryo implantation and early pregnancy maintenance, as it provides a receptive microenvironment that supports trophoblast invasion, placental development, and immune tolerance.^[^ ^3^, ^4^ ^]^ At the same time, the ovary regulates the cyclic secretion of key steroid hormones—primarily estrogen and progesterone—that guide follicular maturation and govern endometrial proliferation, differentiation, and shedding.^[^ ^5^ ^]^ Any disturbance in ovarian hormone synthesis or in endometrial sensitivity may result in infertility, failed implantation, or loss of pregnancy.^[^ ^6^ ^]^ Given the intricate endocrine crosstalk and temporal coordination required for reproductive success, there is a critical need for physiologically relevant in vitro systems that can faithfully model these complex interactions. In response, a growing body of research has focused on developing sophisticated 3D in vitro assay systems that recapitulate the multicellular architecture and bidirectional communication between the ovary and endometrium.^[^ ^7^, ^8^ ^]^ Nevertheless, existing ovary‐ and uterus‐related organ‐on‐a‐chip (OOC) devices remain challenged by significant limitations that impede their translational and physiological applicability. To address these challenges, we developed a biomimetic ovary–endometrium organ‐on‐a‐chip system that faithfully reconstructs the structural complexity, endocrine interactions, and menstrual cycle dynamics of the human reproductive axis.

First, conventional reproductive OOC systems primarily utilize 2D monolayer or single‐cell cultures, which are inadequate for replicating the complex, extracellular matrix (ECM)‐rich 3D microarchitecture found in native tissues.^[^ ^9^, ^10^, ^11^ ^]^ In vivo, the maintenance of ovarian follicle and endometrial function is heavily reliant on the spatial arrangement and continuous communication among diverse cell types within a highly organized 3D microenvironment.^[^ ^12^, ^13^, ^14^ ^]^ The absence of such architectural and cellular complexity substantially diminishes the physiological relevance of current reproductive OOC platforms for modeling cell behaviors, hormonal responsiveness, and pharmacological effects.

Second, a highly coordinated bidirectional hormonal feedback loop linking the ovary and endometrium is critical for aligning follicular maturation with endometrial receptivity, which is vital for reproductive competence.^[^ ^15^ ^]^ Estrogens and progesterone secreted by the ovary are essential for modulating endometrial proliferation, differentiation, and the establishment of receptivity,^[^ ^16^ ^]^ while prostaglandin E2 derived from the endometrium exerts regulatory effects on ovulatory and luteal activity.^[^ ^17^ ^]^ Nevertheless, the majority of existing reproductive OOC models are restricted to single‐organ representations,^[^ ^18^, ^19^, ^20^, ^21^ ^]^ limiting their capacity to emulate the dynamic, bidirectional, and dose‐dependent endocrine interactions required for female reproductive homeostasis and regulation of fertility.

Third, both the ovary and endometrium exhibit significant cyclical alterations during the menstrual cycle.^[^ ^22^, ^23^ ^]^ Follicular maturation occurs through well‐defined stages (e.g., primary, secondary, and antral follicles),^[^ ^24^ ^]^ and the endometrium sequentially progresses through the proliferative, secretory, and menstrual phases, each defined by distinct structural, cellular, and hormonal features.^[^ ^25^ ^]^ These temporally regulated physiological alterations play a pivotal role in modulating drug response, implantation outcomes, and the pathophysiology of reproductive diseases. Nonetheless, current reproductive OOC models are unable to replicate the temporally dynamic, phase‐specific physiological changes of the ovary and endometrium observed throughout the menstrual cycle.

To overcome the aforementioned limitations, we have established a novel ovary–endometrium organ‐on‐a‐chip system that accurately mimics the 3D organization, cellular diversity, and hormone‐mediated bidirectional signaling between the ovary and endometrium, providing a physiologically relevant representation of the female reproductive axis. Diverse ovarian and endometrial cell populations, alongside endogenous ECM components such as collagen and hyaluronic acid, were deliberately introduced to recreate a physiologically representative 3D tissue milieu notable for architectural complexity and multicellular diversity. Moreover, each chamber within the device is designed to reproduce stage‐specific structures—such as primary and antral follicular environments in the ovary and follicular versus luteal‐phase endometrial conditions—facilitating real‐time modeling of menstrual cycle transitions. Additionally, the ovarian and endometrial chambers within the organ‐on‐a‐chip system are linked by a media channel lined with endothelialized vasculature, supporting physiologically relevant, bidirectional hormonal interaction that closely emulates the native ovary–endometrium connection.

Conventional cytotoxicity assays typically depend on functional outcomes such as cell viability, apoptosis, or proliferation, often necessitating extended exposure to high concentrations of toxicants to generate detectable effects.^[^ ^26^ ^]^ However, these terminal functional alterations generally become evident only after several days, thereby restricting both the temporal resolution and sensitivity of these methodologies. Recent studies, however, indicate that transcriptional activation of toxicity‐associated genes (biomarkers) often arises within hours following exposure, serving as a highly sensitive and early molecular indicator of cellular stress in response to even brief or low‐level toxicant stimuli.^[^ ^27^ ^]^ Grounded in this emerging paradigm, we established that a gene marker‐based toxicity detection system can achieve enhanced temporal resolution and improved sensitivity relative to conventional functional assays. Specifically, transcriptional upregulation of toxicity‐inducible genes such as ANGPTL4 and SERPINB2 was observed in ovarian granulosa/thecal cells and endometrial stem cells, respectively—cell populations central to ovarian and endometrial physiology. Our previous investigations further showed that undifferentiated stem cells are more susceptible to toxicant‐induced damage than their differentiated counterparts, highlighting the significance of stem cell‐based toxicity models for early‐stage detection. In response, we engineered a genetically encoded reporter system by linking the promoter regions of ANGPTL4 and SERPINB2 to fluorescent proteins (GFP or mCherry) and stably integrating these constructs into granulosa cells, thecal cells, and endometrial stem cells. Following a toxicant challenge, activation of these specific promoters induces measurable fluorescent signals, enabling real‐time and non‐invasive monitoring of cellular toxicity. This bioengineered reporter‐based ovary–endometrial organ‐on‐a‐chip platform provides quantitative toxicity assessment based on gene‐level responses, representing a sensitive, early, and physiologically relevant alternative to traditional cytotoxicity assays.

## 2 Results

### 2.1 Fabrication of a Multicompartmental Ovary‐Endometrial Organ‐on‐a‐chip Platform Using 3D Printing

**Figure 1:**
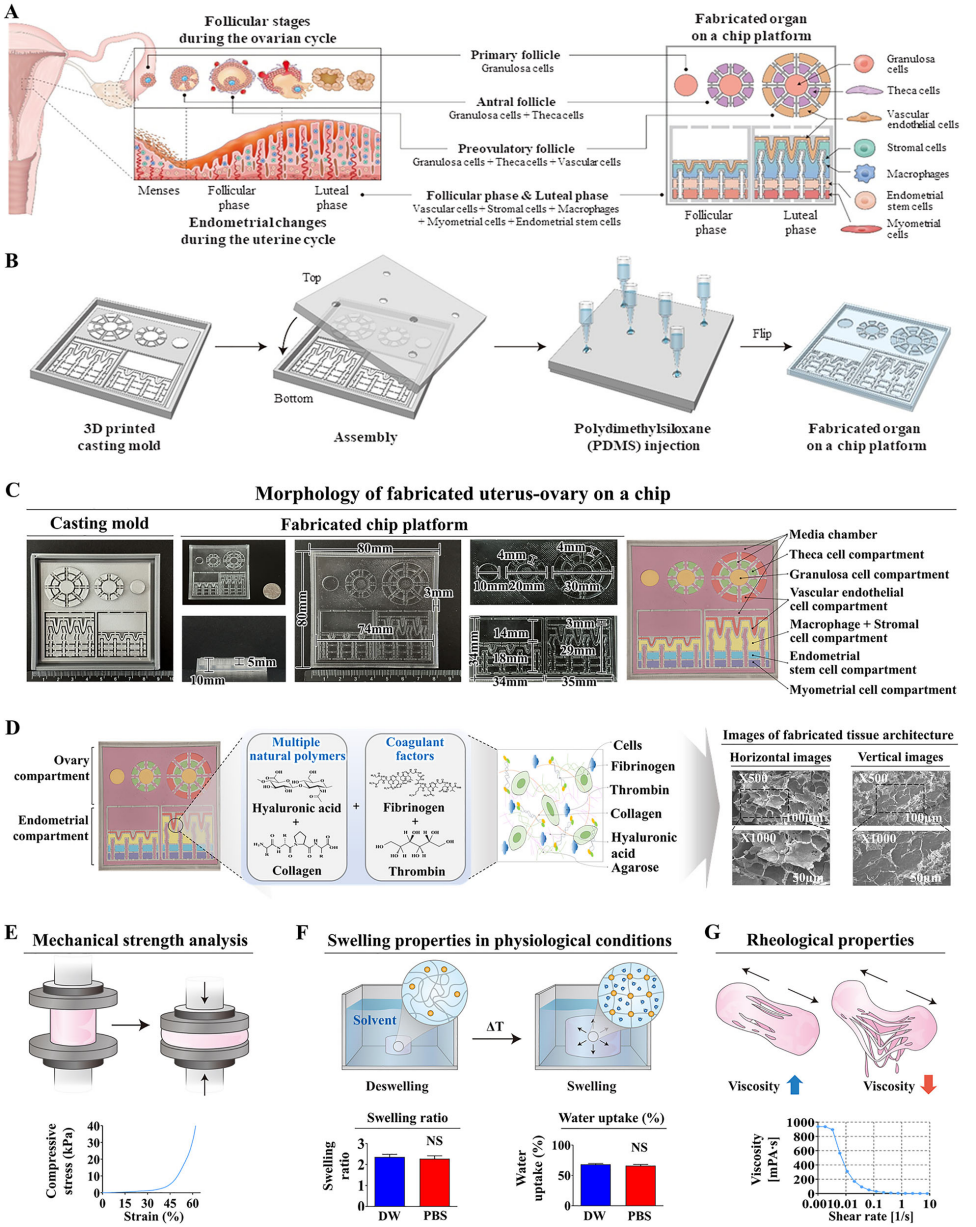
Construction of a multicompartmental ovary–endometrium organ‐on‐a‐chip platform using 3D printing and mechanical evaluation of biomaterial‐based tissue structures. Schematic illustration of the human ovary–endometrium organ‐on‐a‐chip device, engineered to reproduce essential structural and functional characteristics of the menstrual cycle. The ovary compartment consists of anatomically distinct follicular regions—primary, antral, and preovulatory follicles—each housing granulosa, theca, and vascular endothelial cells specific to each developmental stage. The endometrial compartment simulates the follicular and luteal phases, incorporating endometrial stem cells, stromal fibroblasts, myometrial cells, macrophages, and vascular cells positioned in physiologically relevant arrangements. A surrounding media chamber enables endocrine signaling between the compartments, mirroring in vivo hormonal interactions A). Workflow showing the process of casting mold fabrication via high‐resolution 3D printing. CAD‐based models were translated into 3D‐printed casting molds using poly (lactic acid) (PLA), followed by PDMS injection and thermal curing to produce a structurally integrated elastomeric device B). Images showing the 3D‐printed mold (left), the polymerized PDMS‐based organ‐on‐a‐chip device (center), and the assembled chip platform (right). The compartments for specific cell types and media chambers are strategically organized to replicate anatomical configuration and physiological signal transmission routes C). SEM image depicting the porous ultrastructure of the natural polymer‐based 3D tissue architecture, composed of collagen, hyaluronic acid, fibrinogen, and thrombin, to promote cell adhesion and viability D). Uniaxial compression testing of the natural polymer‐based tissue construct demonstrates an average compressive strength of ∼40 kPa, supporting its mechanical suitability for physiological environments E). Assessment of scaffold swelling under physiological conditions (37 °C, pH 7.4) in distilled water and PBS indicates minimal deformation and stable structural integrity of the engineered tissue architecture F). Rheological assessment of the tissue architecture demonstrates a shear‐thinning profile, with dynamic viscosity decreasing from ∼1000 Pa·s to near zero as the shear rate rises from 1 to 10 s^−1^, highlighting suitable viscoelastic properties for maintaining cellular viability and function G).

To accurately reproduce the complex hormonal interactions and multicellular organization of the human female reproductive system, we established a biomimetic ovary–endometrial organ‐on‐a‐chip (OOC) platform that both structurally and functionally replicates essential features of in vivo physiology. The platform incorporates several advanced elements enabling dynamic modeling of ovarian–uterine interactions throughout the menstrual cycle. First, the ovarian and endometrial compartments are linked via a medium channel lined with vascular endothelial cells, effectively mimicking the bidirectional steroid hormonal exchange observed between the ovary and endometrium in vivo. Second, to capture the changing stages of the menstrual cycle, the ovarian chamber is divided into three anatomically defined follicular zones: primary follicle, antral follicle, and preovulatory follicle regions, each seeded with granulosa and theca cells at their respective developmental phases. Concurrently, the endometrial chamber is organized to reflect both follicular and luteal cycle phases, with specific cellular compositions and tissue structures mirroring hormone‐regulated cyclical changes in the endometrium. Third, the platform includes a broad spectrum of cell types that define the reproductive microenvironment. The ovarian compartment is composed of granulosa cells, theca cells, and vascular endothelial cells—each essential for follicle development and steroid hormone synthesis. The endometrial chamber was populated with five physiologically relevant cell types, each selected to mimic distinct structural and functional layers of the uterine lining: endometrial stem cells (representing the regenerative basal layer), stromal fibroblasts (mimicking the mesenchymal stroma), vascular endothelial cells (lining the microvasculature), uterine macrophages (modeling resident immune surveillance), and myometrial smooth muscle cells (representing the contractile outer layer) (**Figure** **1**A).

To capture the rapid and precise morphological alterations of both the ovary and endometrium during the menstrual cycle, we applied high‐resolution 3D printing technology to fabricate a biologically specific mold for the OOC device. This enabled micron‐level accuracy in the development of microchannels representing each reproductive cell compartment. After designing the mold, polydimethylsiloxane (PDMS) was injected and thermally cured within it, resulting in the final elastomeric chip structure (Figure 1B).

In Figure 1C, the left panel shows the 3D‐printed casting mold utilized for creating the PDMS‐based organ‐on‐a‐chip device. This mold served as a high‐precision negative form into which PDMS was introduced and then thermally set, yielding the finished chip as depicted in the central panel. The right panel in Figure 1C demonstrates the spatial arrangement of microchannels within the platform, where each channel is designated for specific cell types and their relevant stages in the menstrual cycle.

### 2.2 Evaluation of Key Mechanical and Architectural Properties in Natural Polymer‐Based 3D Tissue Constructs Incorporated within the Organ‐on‐a‐Chip System

All cell types were encapsulated within a composite hydrogel formed from natural polymers (collagen and hyaluronic acid), aiming to accurately recapitulate the 3D tissue microenvironment. Given the well‐documented mechanical shortcomings of natural hydrogels, we incorporated the endogenous coagulation factors fibrinogen and thrombin as biocompatible crosslinkers, which enhanced the physical integrity and gelation kinetics of the matrix while maintaining low cytotoxicity. To confirm the structural appropriateness of the ECM‐mimetic hydrogel scaffold for supporting multicellular assemblies, we utilized scanning electron microscopy (SEM) to visualize the microarchitecture established by natural polymers in conjunction with blood‐derived coagulation factors. (Figure 1D). A longstanding challenge in tissue engineering with natural polymers is achieving adequate mechanical robustness to maintain structure under physiological hydration.^[^ ^28^, ^29^ ^]^ In response, we assessed whether the engineered tissue constructs demonstrated essential mechanical features, such as compressive strength, swelling behavior, and rheological properties. To characterize the mechanical performance of the natural polymer‐based 3D tissue construct integrated within the organ‐on‐a‐chip platform, we conducted a uniaxial compressive stress test—a standard approach for determining load‐bearing properties of engineered soft tissues. As depicted in Figure 1E, the composite hydrogel scaffold registered an average compressive stress of nearly 40 kPa, signifying adequate stiffness to retain structure under physiologically relevant conditions. We also determined the construct's swelling properties under physiological conditions by measuring fluid uptake in both distilled water and PBS at 37 °C and pH 7.4. The findings indicated minimal changes in the construct's overall volume and morphology following fluid absorption under these conditions (Figure 1F). The viscoelastic characteristics of natural polymer‐based tissue constructs are critically important in governing their mechanical stability, thereby influencing the viability and function of encapsulated cells.^[^ ^30^ ^]^ Although biomaterials with low viscosity may exhibit compromised structural fidelity due to their highly hydrated and gelatinous nature, they can nevertheless improve the survival of encapsulated cells by creating a permissive and hydrated matrix environment.^[^ ^31^ ^]^ To assess the viscoelastic properties of the engineered tissue constructs, we determined the dynamic viscosity across a range of shear rates. When the shear rate increased from 1 to 10 s^−1^, the viscosity showed a pronounced decrease from ≈1000 Pa·s to near‐zero (Figure 1G). Together, these findings demonstrate that the fabricated 3D tissue construct provides a physiologically relevant microenvironment that supports and sustains multiple cellular populations characteristic of the ovary and endometrium.

### 2.3 Assessment of Long‐Term Cell Viability and Metabolic Activity in Encapsulated Ovarian and Endometrial Cells Using Natural Polymer‐Based Tissue Architecture Within a Chip Platform

To populate the ovarian compartments of the chip, two major types of hormone‐producing follicular cells—granulosa and theca cells—were obtained from patients undergoing in vitro fertilization (IVF). In particular, freshly isolated human theca (Figure S1A, Supporting Information) and granulosa cells (Figure S2A, Supporting Information) were collected from follicular aspirates during IVF procedures, using methodologies previously described.^[^ ^32^ ^]^ To confirm the functional properties of the isolated human theca and granulosa cells, we analyzed the expression of receptors involved in follicular development and steroid hormone synthesis. Western blotting (Figures S1B and S2B, Supporting Information) and immunocytochemistry (Figures S1C and S2C, Supporting Information) demonstrated the presence of follicle‐stimulating hormone receptor (FSHR) and luteinizing hormone receptor (LHR), both essential for gonadotropin‐mediated signaling in theca cells. Furthermore, the estrogen receptor (ER) and progesterone receptor (PR)—key regulators of steroid hormone feedback and ovarian function—were also identified through immunocytochemical analysis (Figures S1C and S2C, Supporting Information), supporting the endocrine capability of these cells. To determine whether theca cells maintained the ability to produce steroid hormones in response to hormonal cues, we treated them with FSH and LH and then measured progesterone, pregnenolone, and estrogen concentrations in the culture medium via ELISA (Figures S1D and S2D, Supporting Information), which demonstrated that both theca and granulosa cells preserved their functional activity and hormonal responsiveness in vitro. Because these cells have been widely used as vascular models in vitro,^[^ ^26^ ^]^ human umbilical vein endothelial cells (HUVECs) were chosen as the primary endothelial cell type in this investigation. Morphologically, HUVECs displayed the characteristic polygonal shape (Figure S3A, Supporting Information) and stained positively for established endothelial markers such as platelet endothelial cell adhesion molecule‐1 (PECAM‐1) and von Willebrand factor (vWF), as confirmed by immunostaining (Figure S3B,C, Supporting Information). Human endometrial stem cells were effectively isolated and extensively expanded using a refined collagenase‐based enzymatic digestion protocol previously developed by our group^[^ ^33^ ^]^ (Figure S4A, Supporting Information). To verify the stemness characteristics of these isolated cells, flow cytometry was conducted to examine the expression of a comprehensive panel of well‐established positive (e.g., CD44, CD73, CD105, CD140b, CD146, and W5C5) and negative (e.g., CD34 and CD45) surface markers (Figure S4B, Supporting Information). Additionally, their multipotent differentiation ability was examined by successful induction into adipogenic and osteogenic lineages, demonstrated by lipid droplet formation (Oil Red O staining, Figure S4C, Supporting Information) and calcium matrix deposition (Alizarin Red S staining, Figure S4D, Supporting Information), respectively. To replicate the endometrial stromal cell population in the organ‐on‐a‐chip system, we utilized human dermal fibroblasts as a functional alternative. Dermal fibroblasts were chosen owing to their common mesodermal origin and notable phenotypic and functional resemblance to native endometrial stromal cells.^[^ ^34^ ^]^ Morphological assessment demonstrated that these cells exhibited a typical spindle‐like morphology (Figure S5A, Supporting Information). Immunocytochemistry analysis further verified their mesenchymal phenotype, showing strong vimentin positivity and absence of cytokeratin 14 (CK14) expression (Figure S5B,C, Supporting Information), aligning with a fibroblast‐like profile. Human macrophages served as a model for tissue‐resident immune cells in reproductive tissues. These macrophages displayed a characteristic round shape, as shown in Figure S6A (Supporting Information), and were verified by immunoreactivity for the myeloid lineage markers CD11b and CD68 (Figure S6B,C, Supporting Information), which confirmed both their identity and activation state. As a further uterine cell type, human myometrial cells—sourced from the outer muscular layer of the endometrium—were identified morphologically by their spindle‐shaped structure (Figure S7A, Supporting Information), and further validated using positive immunofluorescence for muscle‐specific proteins MyoD (Figure S7B, Supporting Information) and myogenin (Figure S7C, Supporting Information).

**Figure 2:**
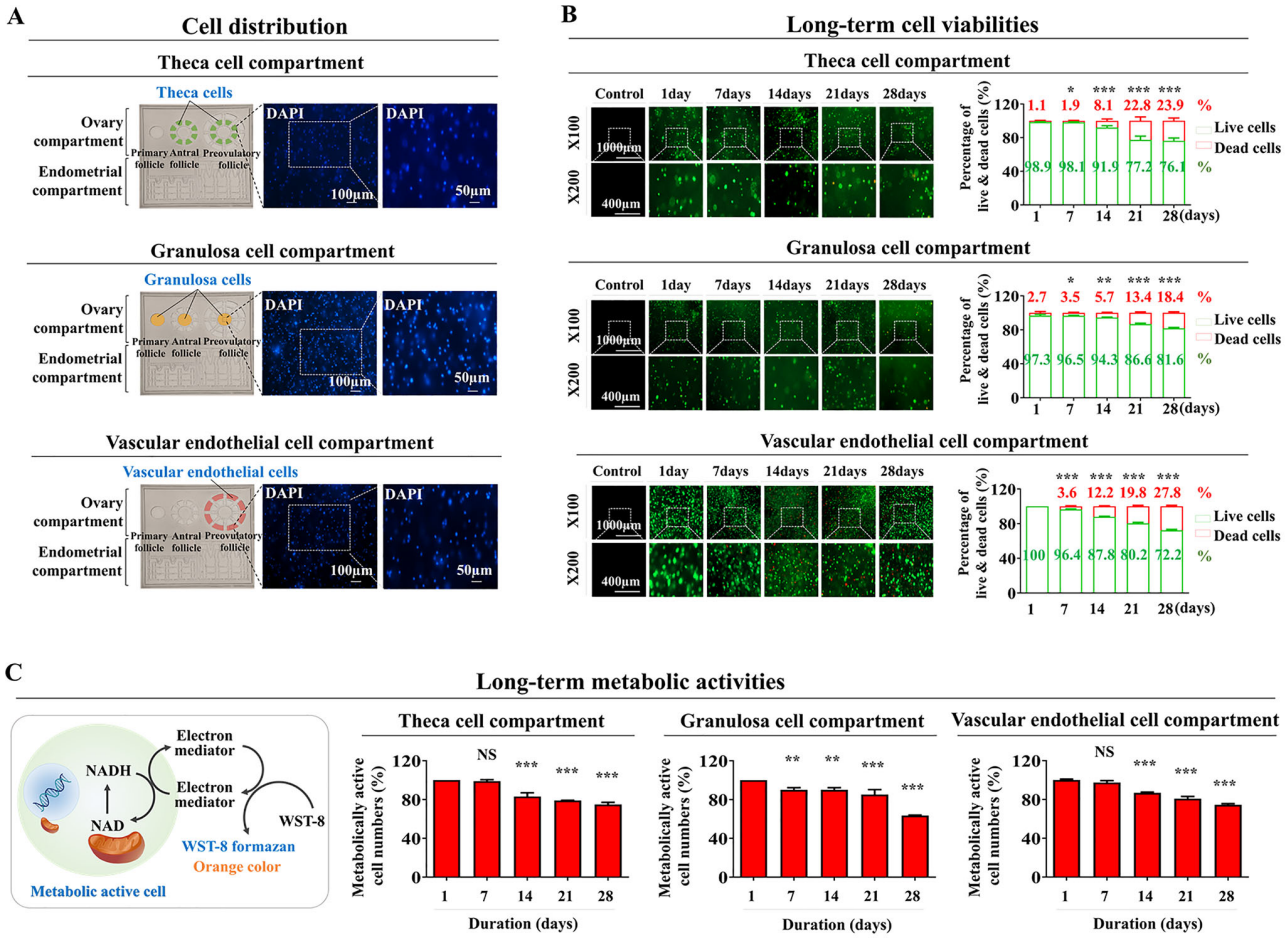
Long‐term viability and metabolic activity of ovarian cell types encapsulated within the ovarian compartments of the organ‐on‐a‐chip microenvironment. Representative nuclear staining of granulosa, theca, and vascular endothelial cells in their corresponding ovarian compartments demonstrates even distribution of encapsulated cells among follicular stages within the natural polymer‐based 3D matrix, reflecting consistent spatial localization and efficient incorporation of multiple ovarian cell types within the engineered tissue construct A). Live & Dead assays were performed on days 1, 7, 14, 21, and 28 after encapsulation to monitor longitudinal viability of each ovarian cell type within their respective compartments on the organ‐on‐a‐chip platform. Quantitative analysis of viability revealed sustained cell survival across 28 days, with encapsulated granulosa cells, theca cells, and vascular endothelial cells displaying >95% viability at early intervals and >70% at day 28 B). CCK‐8 assay results quantifying metabolic activity indicated that despite progressive decline, each encapsulated ovarian cell population preserved over 70% metabolic function at day 28, confirming the biocompatibility and supportive nature of the natural polymer‐based tissue scaffold C). DAPI (blue) counterstains nuclei. Scale bars: 50 µm, 100 µm, 400 µm, and 1000 µm. Data are presented as mean ± SEM; ^*^ *p* < 0.05, ^**^ *p* < 0.01, ^***^ *p* < 0.001.

**Figure 3:**
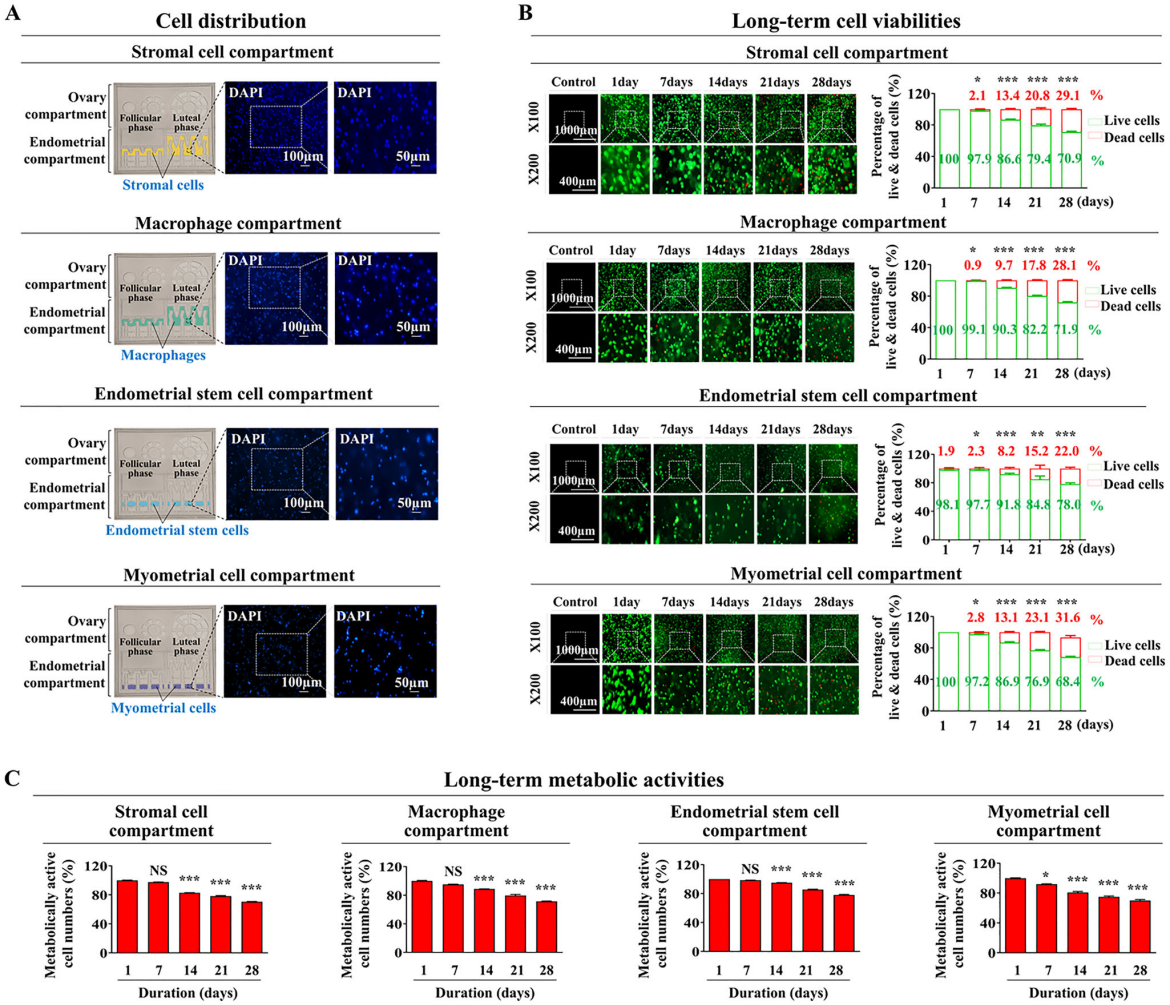
Sustained viability and metabolic activity of various endometrial cell types encapsulated within the endometrial compartment of an organ‐on‐a‐chip platform. All four cell types (endometrial stem, stromal cells, macrophage, and myometrial cells) are incorporated into the natural polymer‐based tissue matrix with defined spatial arrangements. Representative nuclear staining images of encapsulated endometrial cell types show uniform distribution throughout the follicular and luteal phase‐mimicking compartments A). Longitudinal Live & Dead assay outcomes display continuous cell viability for all endometrial cell types from day 1 to day 28, achieving survival rates above 70% at the final evaluation B). Results from the CCK‐8 assay indicate a gradual reduction in metabolic activity over time, yet all endometrial cell populations retained considerable metabolic function through day 28, demonstrating the adequacy of the natural polymer‐based tissue microenvironment for extended in vitro culture C). DAPI (blue) counterstains nuclei. Scale bars: 50, 100, 400, and 1000 µm. Data are presented as mean ± SEM; ^*^ *p* < 0.05, ^**^ *p* < 0.01, ^***^ *p* < 0.001.

Various combinations of natural polymers were utilized to encapsulate these established ovary‐ and endometrium‐derived cell populations, which were then positioned within their anatomically designated compartments in the chip platform. To determine whether the encapsulated cells were evenly distributed throughout the natural polymer‐based 3D tissue structures, we conducted nuclear staining on cells placed in each compartment. As demonstrated in **Figures** **2**A and 3A, the nuclei appeared uniformly distributed within the 3D tissue matrices, confirming even spatial organization of multiple ovarian (theca cells, granulosa cells, and vascular endothelial cells) and endometrial (stromal cells, macrophages, endometrial stem cells, and myometrial cells) cell types in the chip platform. Achieving such uniformity is essential for maintaining consistent cellular communication, nutrient availability, and reliability in downstream biological analyses. Next, we examined long‐term cellular viability of the encapsulated cells by performing the Live & Dead assay on days 1, 7, 14, 21, and 28. While a gradual decrease in cell survival was observed over time, a considerable proportion of both ovarian (Figure 2B) and endometrial (**Figure** **3**B) cells remained viable, with over 70% survival noted at day 28. These results indicate that the natural polymer‐based 3D microenvironment within the chip platform is sufficiently cytocompatible to support prolonged cell viability during extended culture periods. Beyond survival, the long‐term metabolic activity of these cells was assessed via the CCK‐8 assay using identical time points. As shown in Figure 2C and Figure 3C, a sustained but progressive reduction in metabolic activity occurred with longer culture, yet most cells retained more than 70% of their metabolic function up to day 28. These observations demonstrate that the biomimetic 3D tissue environment in the organ‐on‐a‐chip platform establishes a stable niche capable of maintaining both cell viability and metabolic functionality over extended durations. Together, these findings affirm the biological compatibility and functional integrity of the natural polymer‐based 3D microenvironment for encapsulating ovarian and endometrial cells in the chip, supporting its application in prolonged in vitro reproductive modeling and toxicity evaluation.

### 2.4 Maintenance of Cell‐Type‐Specific Phenotypes and Functional Properties Within the Natural Polymer‐Based 3D Microenvironment of the Organ‐on‐a‐Chip Platform

**Figure 4:**
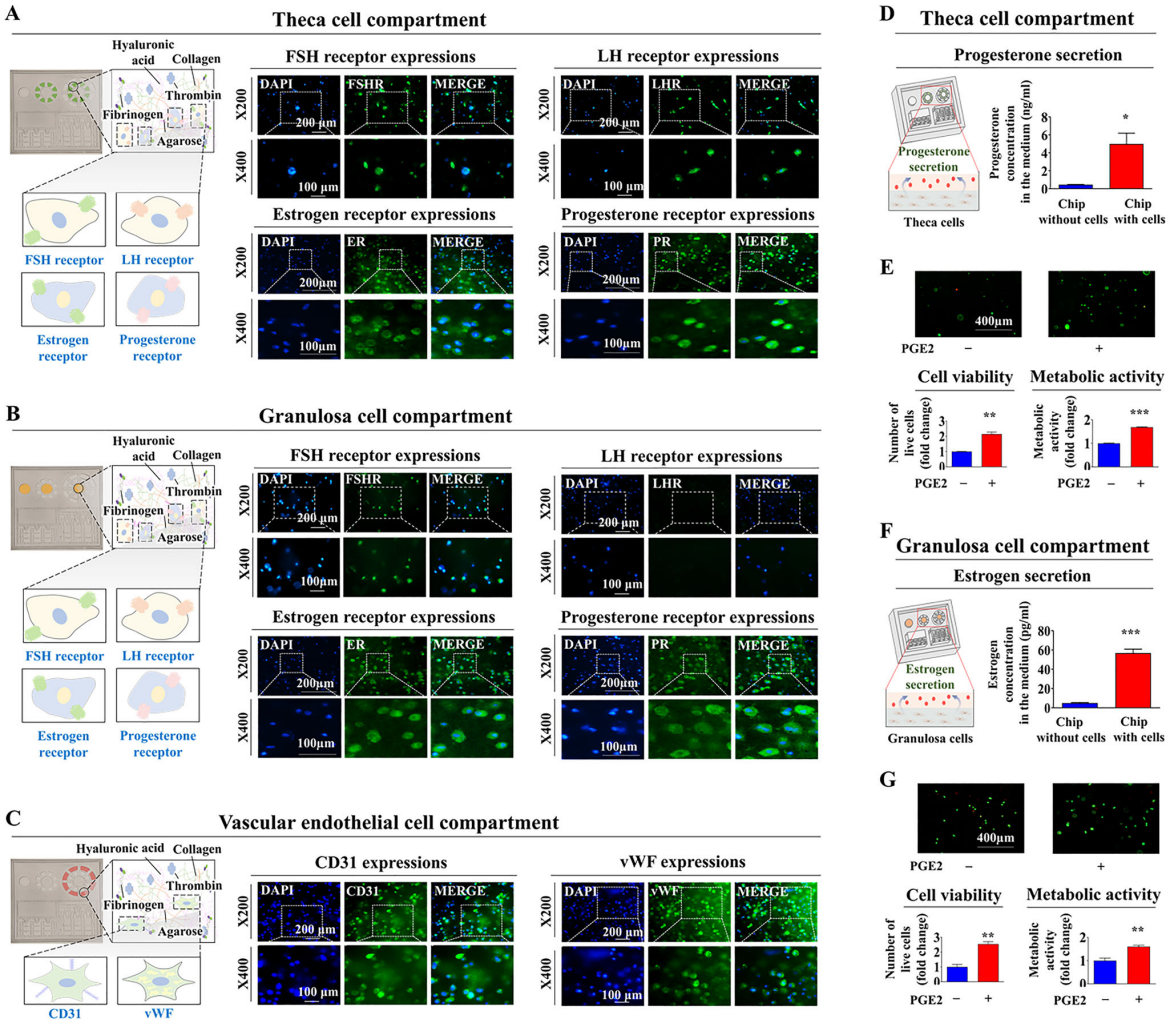
Maintenance of phenotypic identity and functional competence of ovarian cells encapsulated within the natural polymer‐based 3D microenvironment of the organ‐on‐a‐chip platform. Representative immunofluorescence images display strong immunoreactivity for luteinizing hormone receptor (LHR), follicle‐stimulating hormone receptor (FSHR), estrogen receptor (ER), and progesterone receptor (PR) in theca cells housed in the designated theca compartment A). Granulosa cells visualized within the granulosa compartment exhibit robust expression of FSHR, ER, and PR, with only weak LHR signal, thereby mirroring their in vivo hormone receptor distribution B). Vascular endothelial cells situated in the microvascular compartment adjacent to the ovary chamber show maintained angiogenic characteristics, as evidenced by positive staining for CD31 (PECAM‐1) and von Willebrand factor (vWF), confirming retention of endothelial phenotype within the natural polymer‐based 3D tissue matrix C). Progesterone secretion by encapsulated ovarian theca cells was evaluated using ELISA. High levels of progesterone produced by these theca cells indicate sustained steroidogenic function in the organ‐on‐a‐chip system D). A CCK‐8 assay demonstrated increased metabolic activity of theca cells following PGE2 treatment, indicating preserved functional hormonal responsiveness to paracrine signals originating from the uterus E). Estrogen secretion from encapsulated granulosa cells was measured via ELISA, supporting ongoing steroidogenic function in the organ‐on‐a‐chip microenvironment F). Exposure to PGE2 significantly elevated metabolic activity in embedded granulosa cells, as analyzed by CCK‐8 assay, indicating continued responsiveness to paracrine hormonal signals G). DAPI (blue) was used for nuclear counterstaining. Data are presented as mean ± SEM; ^*^ *p* < 0.05, ^**^ *p* < 0.01, ^***^ *p* < 0.001.

To determine if the encapsulated ovarian cells maintained their tissue‐specific phenotypes within the biomaterial‐based 3D microenvironment, we evaluated the expression of key molecular markers specific to each cell type within the defined compartments of the organ‐on‐a‐chip platform. In the theca cell compartment, human theca cells showed robust expression of FSHR, LHR, ER, and PR (**Figure** **4**A), confirming the preservation of their phenotypic identity within the natural polymer‐based 3D tissue context, consistent with their characteristics in native ovarian tissue. Likewise, granulosa cells encapsulated in the ovarian compartment displayed strong expression of FSHR, ER, and PR, whereas LHR expression was comparatively low (Figure 4B), consistent with their receptor profile observed in vivo. In the vascular channel adjacent to the ovarian compartment, vascular endothelial cells similarly preserved their phenotype within the natural polymer scaffold. Specifically, they exhibited strong immunoreactivity for PECAM‐1 and vWF (Figure 4C), which aligns with their angiogenic profile and vascular stability within the 3D microenvironment. Beyond maintaining tissue‐specific phenotypes, we next investigated whether the encapsulated ovarian cells within the biomaterial‐based 3D microenvironment retained functional activity. More specifically, we evaluated their capacity to synthesize steroid hormones and respond to hormonal stimulation—two essential physiological roles of ovarian follicular cells. As demonstrated in Figure 4D,F, both theca and granulosa cells secreted physiologically relevant concentrations of progesterone and estrogen, respectively, indicating that the 3D microenvironment supports steroidogenic activity on par with what is observed in vivo. To further assess the functional responsiveness of these cells to the uterine‐derived hormone PGE2, we measured cell viability and metabolic activity as indicators of functional activation after PGE2 stimulation. As presented in Figure 4E,G, both measures were significantly elevated following PGE2 treatment, suggesting that the encapsulated theca and granulosa cells maintained hormone‐sensing properties and remained functionally responsive within the organ‐on‐a‐chip platform.

**Figure 5:**
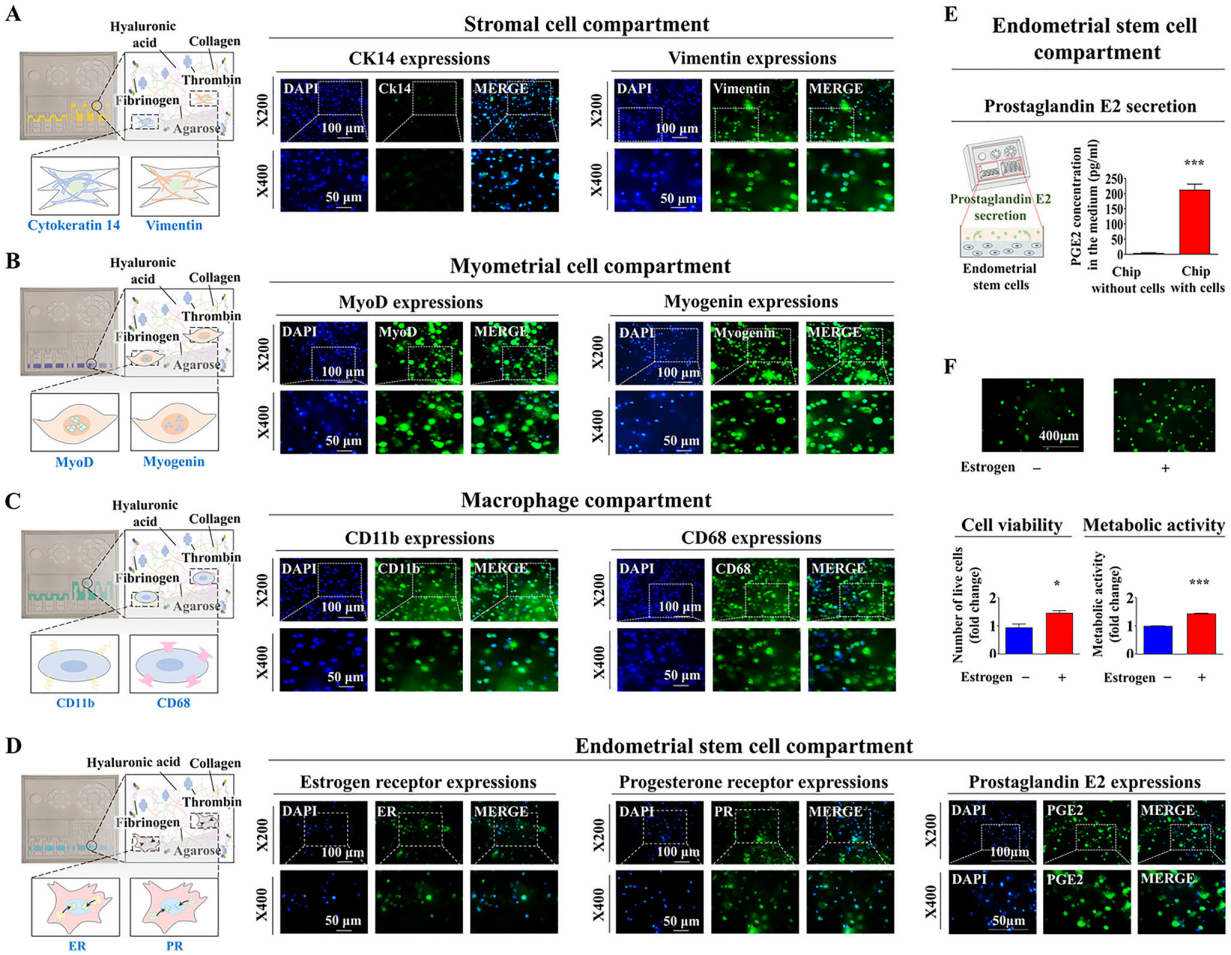
Maintenance of phenotypic characteristics and functional validation of encapsulated endometrial cell types within the natural polymer‐based 3D microenvironment of the organ‐on‐a‐chip platform. Stromal cell compartment: Immunofluorescence analysis revealed pronounced vimentin expression, a canonical mesenchymal marker, while cytokeratin 14 was minimally detected, verifying both the mesenchymal phenotype and the exclusion of epithelial characteristics in encapsulated stromal cells A). Myometrial cell compartment: Embedded myometrial cells demonstrated strong nuclear localization of MyoD and myogenin, two pivotal transcription factors essential for smooth muscle specification and contractile activity B). Macrophage compartment: Immunolabeling for CD11b and CD68 confirmed successful maintenance of the monocytic/macrophage phenotype in encapsulated immune cells, with consistent expression of both markers throughout the different compartments, which supports immunophenotypic stability C). Endometrial stem cell compartment: Endometrial stem cells preserved hormone receptor profiles, exhibiting robust immunoreactivity for estrogen receptor (ER), progesterone receptor (PR), and prostaglandin E2 (PGE2) receptor within the natural polymer‐based 3D tissue structure. These markers endorse physiological responsiveness to steroids in the chip setting D). ELISA‐based quantification of PGE2 secretion from the endometrial chip compartment demonstrated significantly elevated levels in chips harboring endometrial cells compared to acellular controls, confirming retained endocrine functionality E). The hormonal responsiveness of endometrial cells was further examined following estrogen administration. Both overall cell viability and metabolic activity increased substantially in response to estrogen, suggesting intact hormone‐sensing capability within the biomimetic microenvironment F). DAPI (blue) served as a nuclear counterstain. Data are presented as mean ± SEM; ^*^ *p* < 0.05, ^**^ *p* < 0.01, ^***^ *p* < 0.001.

Within the endometrial stromal compartment, encapsulated human stromal cells demonstrated strong vimentin expression, a recognized mesenchymal marker, along with minimal CK14 expression, an established marker indicating a non‐epithelial lineage (**Figure** **5**A). This suggests that the cells maintained their inherent stromal characteristics within the 3D natural polymer‐based microarchitecture. In the myometrial cell compartment, the encapsulated myometrial cells showed pronounced immunoreactivity for MyoD and myogenin, both of which are essential transcription factors implicated in smooth muscle differentiation and contractile capacity (Figure 5B). Likewise, macrophages incorporated within the immune compartment maintained their characteristic immunophenotype, as evidenced by robust expression of CD11b and CD68—widely accepted markers of monocyte/macrophage lineage (Figure 5C). Within the endometrial stem cell compartment, the cells exhibited high expression levels of estrogen receptor (ER), progesterone receptor (PR), and Prostaglandin E2 (PGE2) receptor, consistent with their expected responsiveness to sex steroids in vivo (Figure 5D). Furthermore, we evaluated whether the endometrial compartment retained essential physiological activities. Specifically, we examined its capacity to secrete PGE2, a principal endometrial hormone, and to respond to estrogen, the principal ovarian‐derived steroid hormone responsible for endometrial remodeling. As depicted in Figure 5E, quantitative ELISA analysis demonstrated active PGE2 secretion in the endometrial compartment. To further substantiate the hormonal responsiveness of the endometrial compartment, we treated the system with exogenous estrogen and subsequently assessed alterations in cell viability and metabolic activity. Following estrogen exposure, we observed a substantial increase in both endpoints (Figure 5F), indicating that the encapsulated endometrial cells maintained their functional sensitivity to steroid hormones within the 3D natural polymer‐based microenvironment. Collectively, these results verify that the organ‐on‐a‐chip platform offers a physiologically pertinent 3D microenvironment, enabling various ovarian and endometrial cell types to sustain their native molecular profiles and functional characteristics.

### 2.5 Identification and Verification of Reliable Biomarkers for Predicting Female Reproductive Toxicity

**Figure 6:**
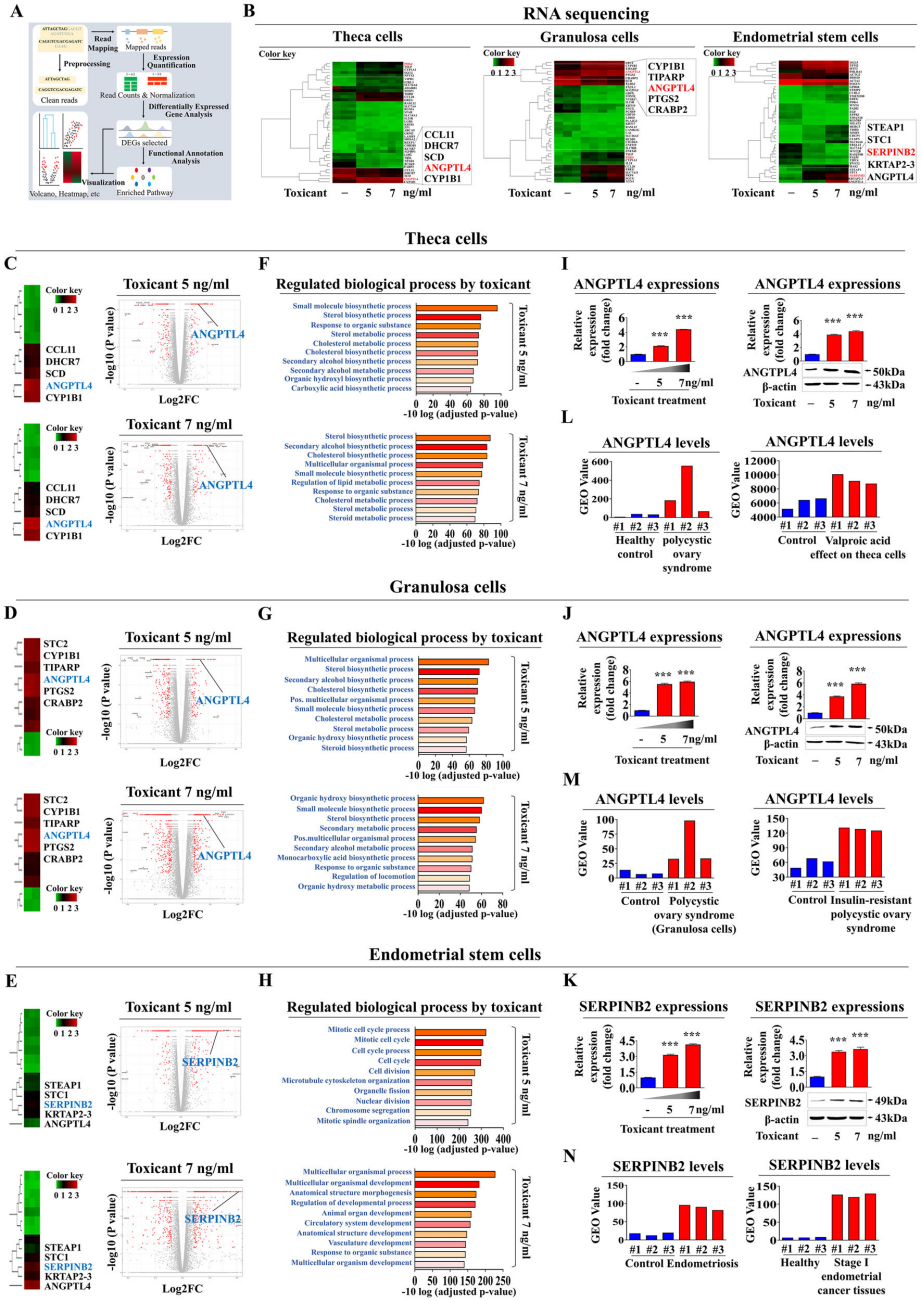
Transcriptomic identification and validation of ANGPTL4 and SERPINB2 as cell‐type‐specific biomarkers for predicting female reproductive toxicity. Schematic workflow illustrating biomarker discovery through a transcriptomics‐driven toxicogenomics strategy. Ovarian theca cells, granulosa cells, and endometrial stem cells were exposed to incremental concentrations (0, 5, 7 ng mL^−1^) of the reference toxicant (dioxin), followed by bulk RNA sequencing to identify differentially expressed genes related to toxicant exposure A). The heatmap depicts distinct transcriptomic signatures among the three cell types in response to reference toxicant. Hierarchical clustering delineates a subset of toxicity‐associated genes, including those implicated in oxidative stress pathways, xenobiotic metabolism, and endocrine disruption B). Of the genes significantly altered by toxicant exposure, ANGPTL4 emerged as the prominently upregulated toxicity‐responsive gene within both theca cells C) and granulosa cells D). In endometrial stem cells, SERPINB2 was identified as the strongly induced gene under identical exposure conditions E). To gain deeper insight into how toxicant exposure modulates intracellular signaling networks in ovarian cells and endometrial stem cells, we conducted Kyoto Encyclopedia of Genes and Genomes (KEGG) pathway enrichment analysis F–H). ANGPTL4 was determined to be the significantly upregulated gene in both theca cells and granulosa cells, displaying a dose‐dependent elevation at both mRNA (qPCR) and protein (Western blotting) levels following the toxicant challenge I.J). Conversely, endometrial stem cells showed strong and dose‐dependent upregulation of SERPINB2, confirmed by qPCR and western blot analysis K). To further substantiate the association between elevated ANGPTL4 or SERPINB2 expressions and toxicant‐induced functional phenotypes, we interrogated publicly available transcriptomic datasets from the Gene Expression Omnibus (GEO) L–N). β‐actin was used as the internal control. Data are presented as mean ± SEM; ^*^ *p* < 0.05, ^**^ *p* < 0.01, ^***^ *p* < 0.001.

Conventional cytotoxicity assays typically rely on delayed functional outcomes such as cell viability or apoptosis, requiring extended exposure times to the toxicant. In contrast, early transcriptional activation of genes related to toxicity can be detected within hours and provides a sensitive measure of cellular stress, even when exposure is minimal or short‐lived. To develop a reliable system for the early detection of molecular toxicity biomarkers, we utilized dioxin as the reference toxicant due to its well‐established nature and international recognition.^[^ ^35^ ^]^ Dioxin was administered to encapsulated cells within an organ‐on‐a‐chip platform, focusing on critical ovarian and endometrial cell populations, including theca cells, granulosa cells, and endometrial stem cells. Dose–response studies were conducted to establish the optimal concentration range for dioxin‐induced cytotoxicity. Each cell type underwent exposure to varying dioxin concentrations, followed by a set of functional assays to evaluate cellular responses. In theca cells (Figure S8A, Supporting Information), granulosa cells (Figure S8B, Supporting Information), and endometrial stem cells (Figure S8C, Supporting Information), we measured dioxin impacts on a spectrum of toxicity‐relevant endpoints, including cell proliferation, induction of apoptosis, activation of caspase‐3 (a central component in apoptotic signaling), cell migration capacity, and expression levels of matrix metalloproteinases (MMP‐2 and MMP‐9). After determining effective dioxin concentrations for each cell population, we conducted bulk RNA‐Seq on theca, granulosa, and endometrial stem cells treated with incremental doses to identify genes exhibiting dose‐dependent transcriptional changes (**Figure** **6**A). This transcriptome analysis uncovered a specific group of differentially expressed genes linked to cellular stress responses and toxicity‐associated pathways in all three cell types (Figure 6B). Tables S1–S3 (Supporting Information) list the differentially expressed genes in human theca cells, granulosa cells, and endometrial stem cells, respectively, in response to toxic exposure. To further refine genes of interest pertinent to female reproductive toxicity, we applied KEGG pathway enrichment analysis, which emphasized gene networks and pathways involved in xenobiotic metabolism, inflammation, and endocrine disruption. Of the genes significantly altered by toxicant exposure, ANGPTL4 emerged as the prominently upregulated toxicity‐responsive gene within both theca cells (Figure 6C) and granulosa cells (Figure 6D). Transcriptomic profiling revealed that ANGPTL4 expression was selectively and markedly induced following dioxin exposure, suggesting its role as an ovary‐compartment‐specific early stress‐response gene. In contrast, within endometrial stem cells, SERPINB2 was identified as the strongly induced gene under identical exposure conditions, as shown in Figure 6E, highlighting its potential as an endometrium‐specific injury response marker. To gain deeper insight into how toxicant exposure modulates intracellular signaling networks in ovarian cells and endometrial stem cells, we conducted Kyoto Encyclopedia of Genes and Genomes (KEGG) pathway enrichment analysis. Importantly, pathway enrichment analysis revealed significant activation of cellular signaling pathways associated with stress response, detoxification, and injury repair following toxicant exposure in theca cells (Figure 6F), granulosa cells (Figure 6G), and endometrial stem cells (Figure 6H). To validate the RNA‐sequencing findings, both qPCR and western blot analyses were conducted across all three cell types. As demonstrated in Figure 6I–J, exposure to increasing concentrations of toxicant resulted in a consistent, dose‐dependent upregulation of both mRNA and protein expression levels of ANGPTL4 in two types of ovarian cells and SERPINB2 in endometrial stem cells. To further substantiate the association between elevated ANGPTL4 or SERPINB2 expressions and toxicant‐induced functional phenotypes, we interrogated publicly available transcriptomic datasets from the Gene Expression Omnibus (GEO). This analysis revealed a consistent upregulation of ANGPTL4 or SERPINB2 across multiple experimental models exposed to toxicity‐related conditions (Figure 6L–N). These findings support that ANGPTL4 and SERPINB2 function as reliable, cell‐type‐specific toxicity biomarkers, where ANGPTL4 is indicative of ovarian follicular toxicity, and SERPINB2 reflects endometrial stress responses.

In the ovarian compartment, theca cells and granulosa cells are the two primary somatic cell types that play indispensable roles in follicular development and hormone synthesis. Given their physiological importance and their potential vulnerability to toxicants, both cell types were selected for transcriptomic profiling. This enabled the identification of ovary‐specific early‐response genes, such as ANGPTL4, with strong biological plausibility and functional impact. In contrast, for the endometrial compartment, only endometrial stem cells were subjected to bulk RNA sequencing. This decision was informed by our previously published findings,^[^ ^36^ ^]^ which demonstrated that undifferentiated endometrial stem cells are significantly more sensitive to environmental toxicants compared to their differentiated counterparts. Thus, to enhance the platform's responsiveness to reproductive toxicants and maximize its translational potential for early detection, endometrial stem cells were prioritized for biomarker discovery. Using this approach, we identified SERPINB2 as an endometrium‐specific toxic stress gene.

**Figure 7:**
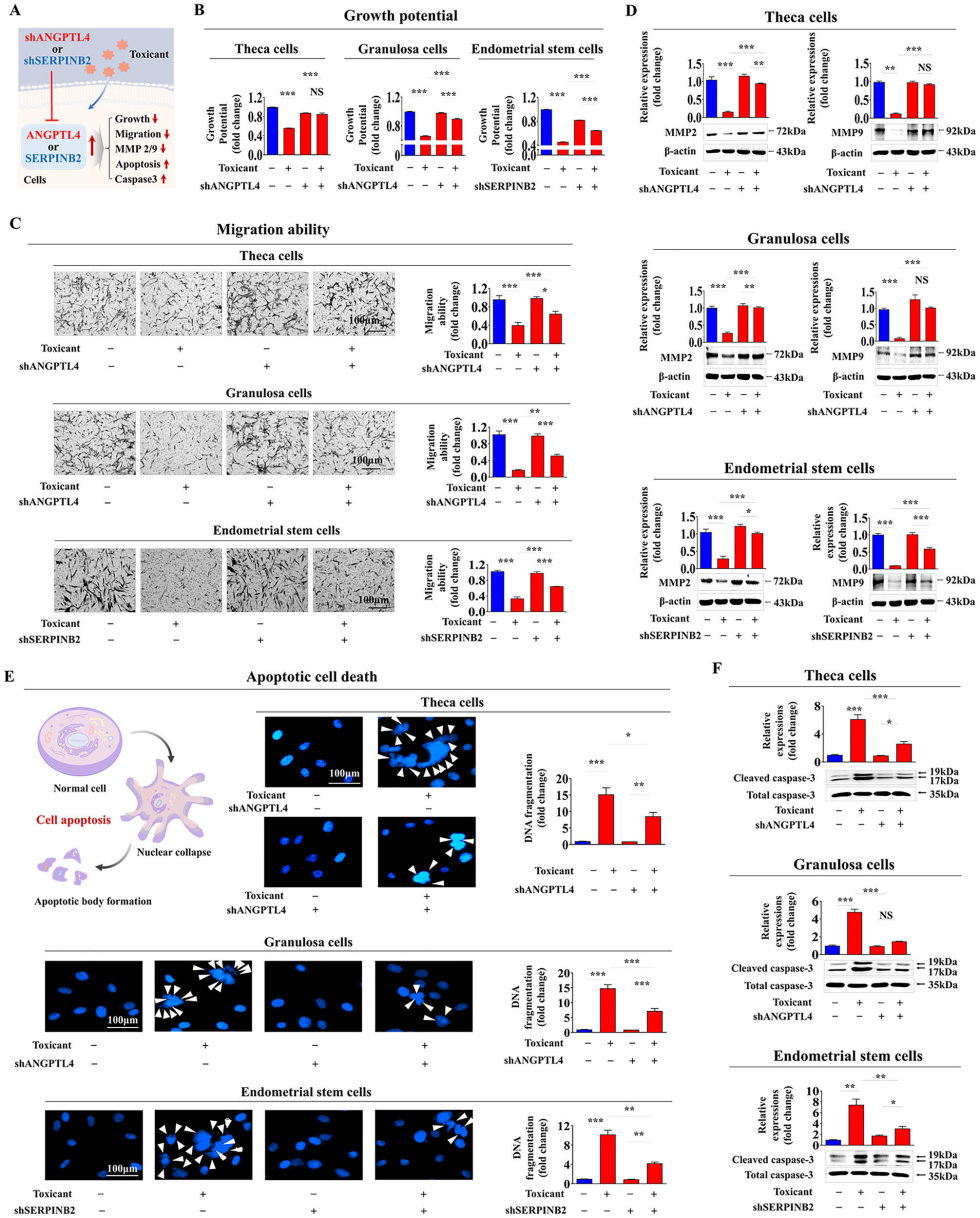
Functional validation of ANGPTL4 and SERPINB2 as key mediators of toxicant‐induced detrimental effects in both ovarian and endometrial cells. The schematic illustrates the experimental approach used to confirm the functional role of ANGPTL4 and SERPINB2 in mediating toxicant‐induced harmful effects in ovarian and endometrial cell populations. Ovarian theca and granulosa cells, together with endometrial stem cells, were transduced with shRNA specifically targeting ANGPTL4 or SERPINB2, followed by treatment with a reference toxicant (dioxin). Multiple facets of cellular toxicity, including alterations in cell growth, migration, and programmed cell death, were then comprehensively evaluated A). Quantitative assessment of cell viability after toxicant exposure, with or without ANGPTL4 or SERPINB2 knockdown, was performed. The toxicant‐mediated reduction in cellular proliferation was significantly mitigated when ANGPTL4 was suppressed in theca and granulosa cells and when SERPINB2 was silenced in endometrial stem cells, highlighting these genes as essential modulators of reproductive toxicity B). Transwell migration assays showed that silencing ANGPTL4 in ovarian cells and SERPINB2 in endometrial stem cells substantially rescued toxicant‐induced deficits in cell motility, pinpointing these genes as key players in toxicity‐associated migration impairments C). Western blot analysis indicated that toxicant‐stimulated increases in matrix metalloproteinase‐2 (MMP‐2) and MMP‐9 expression were significantly diminished by ANGPTL4 silencing in ovarian cells and SERPINB2 knockdown in endometrial stem cells, indicating their involvement in extracellular matrix remodeling following toxicant exposure D). Quantitative analysis of DNA fragmentation characteristic of apoptosis revealed a notable decrease in toxicant‐induced apoptosis in all three cell types when ANGPTL4 or SERPINB2 was silenced, reinforcing their critical contributions to cell death pathways under toxicant stress E). Immunoblot analysis of caspase‐3 activity demonstrated that the toxicant‐driven cleavage of caspase‐3 was significantly reduced by knockdown of ANGPTL4 in ovarian cells and suppression of SERPINB2 in endometrial cells, supporting their role as upstream regulators of apoptosis during toxicant‐induced reproductive toxicity F). DAPI (blue) labels nuclei. β‐actin was used as the internal control. Data are presented as mean ± SEM; ^*^ *p* < 0.05, ^**^ *p* < 0.01, ^***^ *p* < 0.001.

To investigate whether the previously identified biomarkers—ANGPTL4 in two ovarian cell types and SERPINB2 in endometrial cells—act solely as consequence‐driven indicators of toxicant exposure or play a regulatory role in mediating toxicity, we performed targeted gene knockdown experiments. Through shRNA‐mediated silencing, ANGPTL4 and SERPINB2 were individually suppressed in theca cells (Figure S9A, Supporting Information), granulosa cells (Figure S9B, Supporting Information), and endometrial stem cells (Figure S9C, Supporting Information), respectively. After gene knockdown, the cells were exposed to the conventional toxicant, and multiple toxicity‐associated functional outcomes were measured (**Figure** **7**A). We comprehensively assessed the effects of gene knockdown on major toxicity parameters, including proliferation, migration, MMP‐2/9 expression, apoptosis, and caspase‐3 activity. As presented in Figure 7, the depletion of ANGPTL4 or SERPINB2 markedly inhibited the detrimental effects induced by the toxicant across all evaluated outcomes. Toxicant‐induced decreases in cell viability (Figure 7B), migratory ability (Figure 7C), MMP‐2/9 expression (Figure 7D), apoptosis (Figure 7E), and caspase‐3 activation (Figure 7F) were substantially reduced in gene knockdown samples compared to controls. Collectively, these results indicate that ANGPTL4 and SERPINB2 are not simply downstream markers responsive to toxicant exposure, but actively function as key regulators of cellular toxicity in a cell‐type‐dependent fashion.

### 2.6 Development of a Biomarker‐Driven Fluorescent Reporter System for Predicting Reproductive Toxicity in an Organ‐on‐a‐Chip

**Figure 8:**
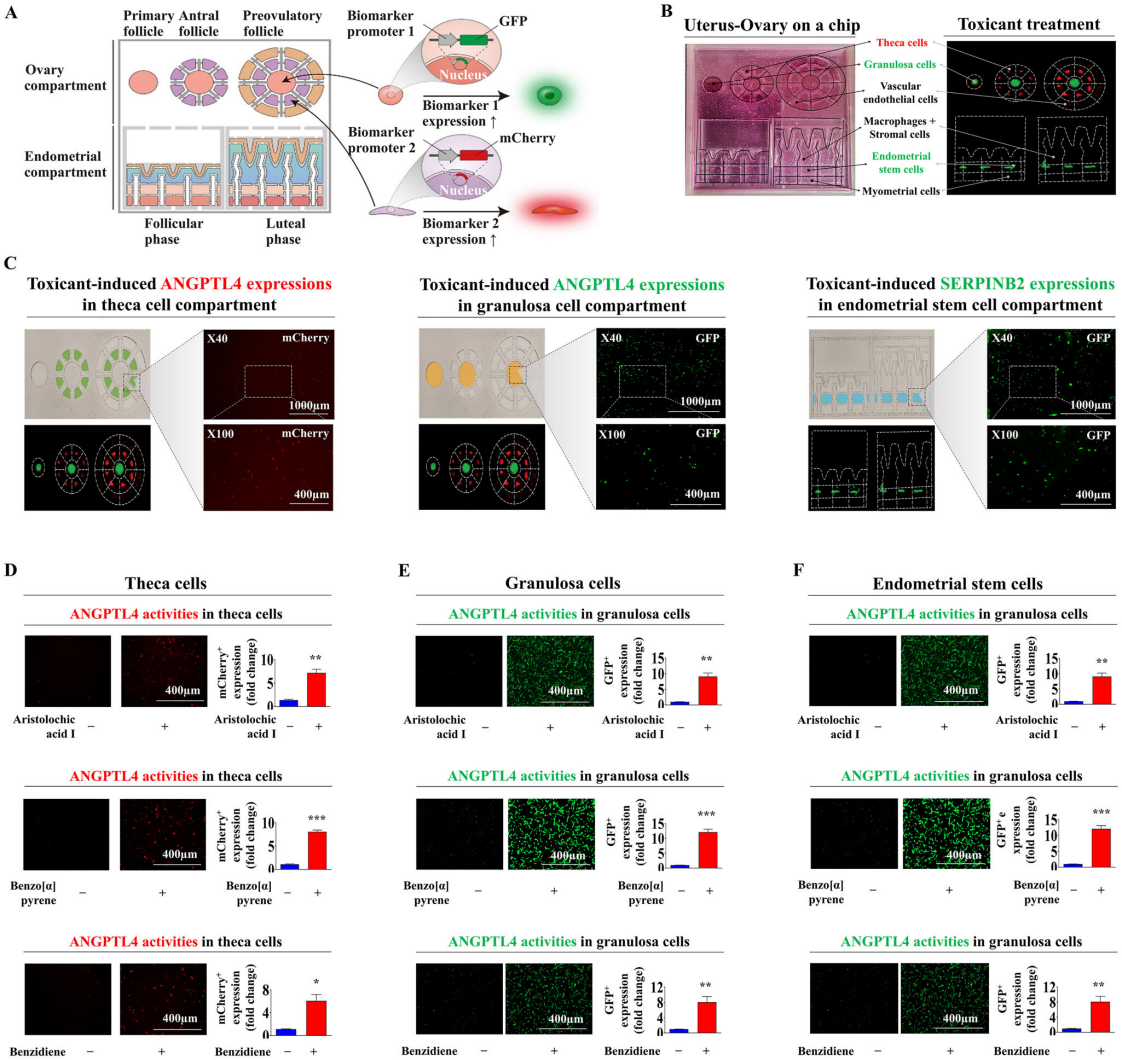
Biomarker‐linked fluorescent reporter system for real‐time prediction of reproductive toxicity in an organ‐on‐a‐chip platform. Schematic diagram of the engineered transcriptional biosensor‐based organ‐on‐a‐chip system. Fluorescent reporter genes were transcriptionally fused to the promoter regions of validated toxicity biomarkers—ANGPTL4 (mCherry in theca cells, GFP in granulosa cells) and SERPINB2 (GFP in endometrial stem cells). These constructs facilitate direct visualization of toxicant‐induced promoter activation within the distinct ovarian and endometrial compartments of the chip A). Representative fluorescence images demonstrate the activation of biomarker‐conjugated fluorescent reporters upon exposure to the reference toxicant (dioxin) B). Theca cells harboring ANGPTL4‐mCherry exhibited red fluorescence, while granulosa cells with ANGPTL4‐GFP showed green fluorescence throughout the primary, antral, and preovulatory follicle compartments. Endometrial stem cells expressing SERPINB2‐GFP displayed green fluorescence following toxicant exposure, verifying cell‐type‐specific promoter activation within the organ‐on‐a‐chip platform C). Quantitative evaluation of mCherry fluorescence driven by the ANGPTL4 promoter in theca cells after treatment with multiple reproductive and genotoxic agents—including aristolochic acid I, benzidine, benzo[α]pyrene, and semustine—showed significant increases in red fluorescence intensity D). Quantitative measurement of GFP expression regulated by the ANGPTL4 promoter in granulosa cells treated with the same array of toxicants revealed robust fluorescence induction for all compounds assessed E). Quantitative analysis of GFP fluorescence regulated by the SERPINB2 promoter in endometrial stem cells after exposure to structurally diverse toxicants demonstrated a substantial induction across all tested agents F). Data are presented as mean ± SEM; ^*^ *p* < 0.05, ^**^ *p* < 0.01, ^***^ *p* < 0.001.

The main objective of this study was to enable fast, intuitive, and quantitative assessment of toxicity responses in reproductive cells using a biomarker‐specific, fluorescent reporter‐based organ‐on‐a‐chip platform. To accomplish this, we constructed a transcriptional biosensing system in which fluorescent reporter genes were placed under the control of promoter regions from toxicity‐associated markers identified in this work (**Figure** **8**A). In particular, the ANGPTL4 promoter region was joined to mCherry in theca cells (Figure S10A, Supporting Information) and to GFP in granulosa cells (Figure S10B, Supporting Information), while the SERPINB2 promoter was linked to GFP in endometrial stem cells (Figure S10C, Supporting Information). These engineered constructs were stably introduced into each target cell line, allowing for direct, real‐time monitoring of toxicant‐induced promoter activity by detecting fluorescent signals (red or green) in a manner specific to each cell type. The biosensing strategy ensured that upon exposure to a toxicant, activation of a biomarker gene would induce expression of the corresponding fluorescent protein, facilitating both qualitative (signal detection) and quantitative (measuring fluorescence intensity) evaluation of cell stress responses inside the chip environment. Following this, the stably modified cells were seeded into their respective areas of the organ‐on‐a‐chip device. To validate the effectiveness of the biomarker‐activated, reporter‐based organ‐on‐a‐chip system, we then exposed chips containing stably transfected ovarian and endometrial cells to the reference toxicant, dioxin. The platform was intended to produce a fluorescent readout upon toxicant‐triggered activation of validated biomarker genes—ANGPTL4 in the ovary and SERPINB2 in the endometrium (Figure 8B). Exposure to the reference toxicant resulted in strong fluorescent reporter signals throughout the ovary compartment, including all follicular subcompartments (primary, antral, and preovulatory follicles). Specifically, both theca cells (expressing ANGPTL4‐mCherry) and granulosa cells (expressing ANGPTL4‐GFP) displayed clearly localized activation of the ANGPTL4 promoter, which was demonstrated by robust red or green fluorescent signals, respectively (Figure 8C). In parallel, endometrial stem cells expressing SERPINB2‐GFP in the endometrial compartment produced prominent green fluorescence following toxicant exposure, indicating active transcriptional induction of SERPINB2 (Figure 8C). To assess whether the engineered biomarker–reporter‐linked screening platform responds solely to dioxin or exhibits broader applicability to different environmental toxicants, we further challenged the system with several toxicants of distinct structures and mechanisms. The compounds included aristolochic acid, benzidine, benzo[α]pyrene, and semustine, each recognized as reproductive or genotoxic agents. Following exposure to each compound, fluorescent reporter activity was assessed in both ovarian and endometrial compartments. As illustrated in Figures 8D–F, each of the tested toxicants led to pronounced activation of fluorescent signals in the transfected cells, demonstrating significant transcriptional activation of the previously validated biomarker genes—ANGPTL4 in theca and granulosa cells, and SERPINB2 in endometrial stem cells. Figure 8D–F focuses on evaluating the universality and specificity of the identified toxicity biomarkers (ANGPTL4 and SERPINB2) in response to multiple toxicants, beyond the standard dioxin challenge. In this context, the critical experimental goal was not to replicate the 3D microenvironment but to assess whether the identified biomarkers respond consistently to different classes of reproductive toxicants (aristolochic acid, benzidine, and benzo[a]pyrene). To facilitate uniform exposure, standardized quantification, and minimize experimental variability, these assays were performed under 2D monolayer culture conditions. Importantly, the fluorescence detected was specific to each compartment and was consistently observed across the various classes of toxicants, indicating that the biomarker–reporter constructs respond to multiple toxic stimuli and are not exclusively reliant on dioxin exposure.

## 3 Discussion

Traditional 2D monolayer cultures do not adequately reproduce the intricate multicellular architecture or the bidirectional endocrine communication that occurs between the ovary and the uterine endometrium,^[^ ^37^, ^38^ ^]^ which restricts their effectiveness for modeling physiologically relevant reproductive processes. In order to overcome the constraints of traditional in vitro culture models for reproductive tissues, we describe the development, fabrication, and functional validation of an innovative fluorescent reporter‐integrated, biomarker‐responsive organ‐on‐a‐chip platform that closely mimics the architectural complexity and physiological interconnectivity observed in the human ovary and endometrium. Human reproductive organs are composed of heterogeneous cell populations interacting through precisely controlled mechanisms that maintain tissue homeostasis and support specialized physiological functions.^[^ ^14^, ^39^, ^40^ ^]^ Unlike previous reproductive organ‐on‐a‐chip approaches, which are typically constrained by simpler designs employing monocultures or limited tissue interaction, our platform integrates a diverse spectrum of primary human cell types—including theca cells, granulosa cells, vascular endothelial cells, endometrial stromal cells, endometrial stem cells, uterine macrophages, and myometrial smooth muscle cells—systematically compartmentalized within biomaterial‐engineered, ECM‐mimicking 3D microenvironments that reflect the spatial and functional diversity found in native reproductive tissues (Figure 1A–C). While the current ECM formulation utilized collagen and hyaluronic acid due to their structural relevance, mechanical stability, and compatibility with 3D microfluidic platforms, future work may incorporate fibronectin and laminin—key modulators of cell adhesion and differentiation in reproductive tissues—to enhance biomimicry and tissue specificity. This compartmentalized approach supports organotypic hormone signaling, enables endocrine feedback mechanisms, and facilitates multicellular paracrine communication, thereby collectively reproducing the cyclical physiology of the female reproductive axis with high accuracy. During the primary follicle stage, granulosa cells are the predominant somatic component, forming a single to few layers around the oocyte, while theca cells are not yet clearly differentiated or hormonally active. Therefore, we modeled this stage using granulosa cells alone. In the antral follicle stage, the recruitment of theca cells becomes essential for androgen production, which granulosa cells then convert into estrogen—representing the hallmark endocrine collaboration of mid‐folliculogenesis. Thus, we introduced both granulosa and theca cells in this region. Finally, the pre‐ovulatory follicle is characterized by pronounced vascularization, particularly in the theca layers, to support steroidogenesis and LH surge responsiveness. To replicate this key feature, we incorporated vascular endothelial cells in addition to GCs and TCs. This stratified cellular configuration enables us to emulate distinct follicular stages and their associated hormone dynamics in a physiologically meaningful manner.

Although the endometrial stem cells utilized in this study exhibit strong regenerative capacity and responsiveness to hormonal cues, it is important to acknowledge that luminal and glandular epithelial cells are indispensable components of the native endometrium. These epithelial populations play essential roles in establishing the endometrial barrier, regulating selective permeability, mediating embryo implantation, and participating in reciprocal signaling with stromal cells. The absence of epithelial cells in our current model represents a notable limitation, particularly in the context of modeling implantation or infection‐associated pathophysiology. We recognize this as a critical area for future improvement to further enhance the physiological fidelity of the endometrial compartment.

Despite successfully engineering an ovary–endometrium organ‐on‐a‐chip that recapitulates core aspects of tissue‐specific hormone secretion, paracrine crosstalk, and cyclic remodeling, a key limitation of the current platform lies in the absence of upstream endocrine regulators—namely, the hypothalamus and pituitary gland. These structures orchestrate reproductive function via secretion of GnRH and gonadotropins (FSH and LH), which initiate and modulate ovarian steroidogenesis and, in turn, influence endometrial physiology. As such, their omission restricts the ability of this system to fully replicate the hierarchical hormonal cascade characteristic of the human menstrual cycle. To address this, our group has previously established a bioengineered hypothalamus–pituitary organ‐on‐a‐chip that faithfully reproduces reciprocal neuroendocrine signaling, including pulsatile GnRH release and responsive LH/FSH secretion.^[^ ^41^ ^]^ Building on this foundation, future efforts will focus on integrating this neuroendocrine module with the current ovary–endometrium chip to reconstruct the entire hypothalamic–pituitary–gonadal (HPG) axis in vitro. Such an advanced multi‐organ system would enable mechanistic exploration of endocrine disruptors, menstrual dysregulation, and hormone‐based therapies with unprecedented physiological fidelity.

In addition, human dermal fibroblasts were employed as a stromal surrogate due to their mesenchymal phenotype, accessibility, and consistent behavior in 3D gel‐based culture. However, we acknowledge that native human endometrial stromal cells possess unique transcriptomic profiles and play a central role in mediating hormone‐driven remodeling, decidualization, and immune‐endocrine crosstalk within the endometrium. The absence of native stromal cells represents a limitation of our current platform and may constrain its ability to fully recapitulate endometrial‐specific paracrine signaling and implantation‐associated responses. Future refinements of the model will benefit from the inclusion of primary or immortalized endometrial stromal cells to enhance physiological fidelity and functional relevance.

Recreating the native 3D microenvironment of ovarian and endometrial tissues in vitro continues to present significant difficulties, mainly because of the distinctive biochemical and biophysical attributes of these organs. For example, hyaluronic acid—a glycosaminoglycan that is present at high levels in reproductive tissues but less so in the majority of other somatic tissues—plays a fundamental role in supporting extracellular matrix hydration, viscoelasticity, and cell–matrix interactions.^[^ ^42^ ^]^ The precise incorporation of such organ‐specific biomolecular elements into engineered platforms is crucial for accurately reproducing the structural and functional characteristics of reproductive organs.^[^ ^43^ ^]^ As an essential extracellular matrix constituent, hyaluronic acid supports the structural foundation of ovarian and endometrial tissues.^[^ ^44^, ^45^ ^]^ In addition to its architectural contributions, it regulates key cellular functions—including proliferation, migration, and inflammatory response—thereby aiding in the maintenance of cellular viability and supporting microenvironmental stability within reproductive organ‐on‐a‐chip models. The proportion of collagen is crucial in determining the ultrastructure and mechanical robustness of engineered tissue constructs. Collagen not only provides structural integrity but also influences essential cellular activities—such as fate determination, cell growth, survival, and migration—within the native‐like extracellular matrix environment.^[^ ^46^, ^47^ ^]^ Considering these diverse roles, this study deliberately utilized collagen and hyaluronic acid together to mimic a physiologically authentic 3D microenvironment reflecting both the architectural complexity and mechanical properties of reproductive tissues (Figures 1D–G). Moreover, the excellent biocompatibility of the collagen–hyaluronic acid composite matrix allowed reproductive cells to maintain long‐term viability (Figures 2 and 3) and phenotypic characteristics (Figures 4 and 5), and maintained functional activity (Figure 5E,F) throughout the culture period within the organ‐on‐a‐chip system.

A key innovation of our organ‐on‐a‐chip platform is its dual functionality: it can accurately recapitulate the cellular heterogeneity, tissue‐specific characteristics, and hormone‐responsive behaviors of human ovarian and endometrial compartments while also allowing for early, real‐time, and cell‐type‐specific detection of toxicological responses. Biomarker‐based toxicity screening systems are able to identify cellular responses at earlier phases and with improved precision compared to traditional assays, which predominantly depend on later‐stage functional changes such as alterations in cell proliferation or apoptosis.^[^ ^48^ ^]^ Toxicant‐induced modifications in intracellular signaling pathways and gene expression occur rapidly, often within minutes to hours following exposure.^[^ ^49^ ^]^ However, observable changes in key cellular functions—such as apoptosis or proliferation—usually require several days of incubation before they can be detected.^[^ ^50^ ^]^ Through stable integration of biomarker‐driven fluorescent reporters—including ANGPTL4‐mCherry (red) or GFP (green) in granulosa and theca cells, and SERPINB2‐GFP in endometrial stem cells—our platform enables rapid translation of toxicant‐induced biomarker activation into measurable red or green fluorescence (Figure 8A,B). This approach addresses a significant shortcoming of standard cytotoxicity assays, which mainly rely on delayed phenotypic endpoints, such as apoptosis or viability, that manifest only after sustained or high‐concentration exposures. By using early‐transcriptional response markers, our biosensing solution enables sensitive and rapid detection of toxic effects, including at sub‐cytotoxic concentrations. Consistent with our current findings, previous research by our group has shown that exposure to toxicants or carcinogens markedly induces SERPINB2 expression in human endometrial stem cells,^[^ ^36^ ^]^ umbilical cord blood‐derived stem cells,^[^ ^35^ ^]^ and several populations of cancer stem‐like cells,^[^ ^51^ ^]^ establishing this gene as a conserved molecular responder to cellular stress across multiple stem cell types. Similarly, we have recently demonstrated that ANGPTL4 serves a critical regulatory function in coordinating numerous cytotoxicity‐related cellular processes—such as proliferation, migration, and differentiation—in human endometrial stem cells after exposure to viral^[^ ^52^ ^]^ and bacterial^[^ ^53^ ^]^ antigens. Importantly, suppression of ANGPTL4 or SERPINB2 expression in ovarian theca and granulosa cells, as well as endometrial stem cells, significantly reduced toxicant‐induced loss of cell viability, migratory ability, and abnormal apoptotic responses (Figure 7A–F). In our study, these genes were identified as functionally specific early‐response biomarkers for ovarian and endometrial toxicity based on two key criteria. First, our gene knockdown experiments demonstrated that ANGPTL4 and SERPINB2 are not merely passive markers of cellular stress but actively contribute to modulating toxicant‐induced cellular injury within ovary‐ and uterus‐derived cells, indicating their context‐specific functional roles (Figure 7A–F). Second, both ANGPTL4 (in ovarian cells) and SERPINB2 (in endometrial stem cells) exhibited robust and reproducible upregulation specifically in response to various classes of toxicants, as validated using our cell‐type‐specific organ‐on‐a‐chip compartments (Figure 8D–F). Collectively, these findings indicate that ANGPTL4 and SERPINB2 function as key mechanistic and predictive biomarkers for evaluating toxicity in ovarian and endometrial cells. Moreover, the reliability of this approach was validated by exposing cells to a diverse set of mechanistically distinct reproductive toxicants, including aristolochic acid, benzidine, benzo[α]pyrene, and semustine, each of which elicited robust and compartment‐specific reporter activation (Figure 8D–F). These results highlight the versatility, high sensitivity, and translational relevance of this platform for preclinical reproductive toxicity assessment and mechanism‐driven toxicogenomic research.

Nevertheless, certain limitations persist. First, although the hydrogel‐based 3D environment supports tissue‐like architecture and maintains cell viability over extended periods, it cannot fully emulate the dynamic mechanical forces or fluidic shear stresses that occur in vivo. The integration of active perfusion systems or cyclic hormonal stimulation could further improve physiological relevance. Second, while ANGPTL4 and SERPINB2 were selected as cell‐type‐specific markers of toxic responses, incorporating additional early‐response markers could enhance the platform's diagnostic resolution and broaden its applicability to a wider range of compounds and mechanistic pathways. In designing our toxicity assessment platform, we intentionally adopted a minimalist biomarker selection strategy, assigning one highly responsive and functionally validated marker to each cell type—ANGPTL4 for ovarian theca/granulosa cells and SERPINB2 for endometrial stem cells. This design decision was based on a core principle: to create a high‐confidence, binary‐response platform that minimizes ambiguity in toxicant classification. Incorporating multiple biomarkers within a single cell type, while potentially increasing detection breadth, could introduce interpretational complexity—particularly when different markers yield discordant signals in response to a given toxicant. Such conflicts can undermine the platform's reliability, necessitating arbitrary thresholds or weightings that detract from objectivity and reproducibility. By contrast, the use of a single biomarker per cell type ensures that any observed response—such as fluorescent signal activation via ANGPTL4 or SERPINB2 reporters—can be clearly and confidently attributed to toxicant‐induced cellular injury within the relevant reproductive compartment. This “low false‐positive” approach prioritizes specificity over sensitivity, operating as a conservative filter that only classifies toxicants when clear, mechanistically supported responses are observed. While this may exclude certain toxicants that do not induce ANGPTL4 or SERPINB2, it reduces the likelihood of false signals due to secondary or nonspecific gene induction. We acknowledge that this single‐marker strategy represents a trade‐off, and future iterations of the platform may benefit from incorporating complementary biomarkers (e.g., IL‐1β, CYP family enzymes, oxidative stress markers) with orthogonal mechanistic readouts. Such expansion, however, would require rigorous validation to avoid confounding cross‐talk and to preserve the interpretive clarity of the current system. In this context, our platform may be envisioned as a modular framework, where marker panels can be flexibly adapted to specific use‐cases or toxicant classes while maintaining high interpretability and reproducibility.

While our current ovary–endometrium organ‐on‐a‐chip platform successfully recapitulates local endocrine crosstalk and cyclical hormonal fluctuations driven by ovarian steroidogenesis, it does not directly model the upstream regulation mediated by the hypothalamic–pituitary axis. The absence of brain‐derived hormonal inputs, such as GnRH, FSH, and LH, represents a physiological limitation of the present system. Future iterations of the platform could incorporate exogenous pulsatile GnRH stimulation or sequential FSH/LH treatments to better mimic the hierarchical hormonal control of the reproductive axis and enhance the fidelity of the modeled ovarian cycle. Moreover, utilizing patient‐derived stem cell‐based organoids or iPSC‐derived cell types may increase the potential for personalized disease modeling, offering new pathways for individualized toxicity screening and precision medicine.

In addition, one notable limitation of the present organ‐on‐a‐chip system is the absence of active perfusion and fluidic shear stress, which are key physiological parameters in vivo. In the native female reproductive tract, interstitial flow and vascular perfusion play vital roles in regulating hormone distribution, nutrient exchange, metabolic waste clearance, and mechanotransduction signaling within ovarian and endometrial tissues. The current chip design primarily recapitulates architectural and endocrine dynamics under static conditions, and thus cannot fully reflect the dynamic fluidic microenvironment encountered in vivo. To overcome this limitation, future iterations of the platform could integrate microfluidic perfusion channels with programmable flow rates to emulate physiological shear forces. Incorporation of endothelial‐lined vasculature or interconnected microchannels driven by peristaltic micropumps could enable controlled delivery of exogenous hormones such as GnRH, FSH, and LH, and facilitate real‐time waste removal. Furthermore, applying tunable flow profiles may allow the modeling of cyclic hemodynamic changes across the menstrual cycle, providing a more biomimetic and translationally relevant in vitro system. These improvements will significantly enhance the fidelity of the chip to recapitulate the hormonal crosstalk and tissue homeostasis of the female reproductive axis under near‐physiological conditions

## 4 Conclusion

In conclusion, this study presents a next‐generation, biomarker‐driven reproductive organ‐on‐a‐chip platform that distinctively combines multi‐lineage human primary cells, ECM‐mimetic 3D microenvironments, cyclic reproductive cues, and fluorescent biosensing reporters to provide real‐time, cell‐type‐specific, and mechanistically informative toxicological assessments. By reconstructing the structural, hormonal, and functional complexity of the human ovary–endometrium axis and enabling early detection of toxicant‐induced cellular stress, the system advances beyond the limitations of conventional cytotoxicity assays and previous organ‐on‐a‐chip designs. Although further advancements—such as the addition of endocrine regulatory compartments, expansion of biomarker panels, and introduction of dynamic flow conditions—will further improve physiological accuracy and translational potential, the current platform establishes a solid basis for the evolution of preclinical reproductive toxicity testing, mechanistic toxicogenomics, and personalized risk assessment. Traditional 2D culture systems, though convenient and widely used for toxicity testing, fail to recapitulate the structural and functional complexity of in vivo tissues. Specifically, they lack the spatial architecture, cell–cell interactions, and biochemical gradients required to mimic the dynamic microenvironments of reproductive organs. Similarly, single‐organoid systems, while offering a degree of three‐dimensionality, remain limited in their ability to model inter‐organ hormonal crosstalk—particularly the bidirectional regulation between the ovary and endometrium that is essential for reproductive homeostasis. In contrast, our ovary–endometrium dual‐organ chip provides an integrated microenvironment that supports a compartmentalized yet physiologically interconnected co‐culture of ovarian and endometrial tissues. This platform enables simultaneous monitoring of tissue‐specific toxic responses under coordinated hormonal influence, which is not feasible in isolated or static models. While a direct comparison with 2D or organoid models was beyond the scope of this study, the architectural and functional advantages of our chip highlight its translational potential as a more predictive and physiologically relevant platform for reproductive toxicity screening and mechanistic studies.

## 5 Experimental Section

### Establishment and Immortalization of Human Endometrial Cells and Associated Cell Types

Human endometrial stem cells were isolated from non‐lesional regions of endometrial tissues obtained from uterine fibroid patients under written informed consent, in compliance with protocols approved by the Gachon University Institutional Review Board (IRB No: GAIRB2018‐134). Tissue samples were carefully collected from histologically normal endometrial areas to ensure that isolated cells were not influenced by underlying pathological conditions. The harvested tissues were finely minced and enzymatically digested in Dulbecco's Modified Eagle Medium (DMEM) supplemented with 10% fetal bovine serum (FBS) and 250 U mL^−1^ type I collagenase at 37 °C for 5 h under continuous agitation. Following enzymatic dissociation, the suspension was passed through a 40 µm cell strainer to separate stromal‐like stem cells from glandular epithelial clusters and residual tissue fragments. Isolated human endometrial cells were subsequently cultured using previously validated protocols,^[^ ^54^ ^]^ employing StemPro MSC SFM CTS medium (GIBCO, Cat. No. A1033201) under standard incubation conditions (37 °C, 5% CO_2_). Media were refreshed every two days. Primary human umbilical vein endothelial cells (HUVECs; ATCC PCS‐100‐010) were acquired from ATCC and maintained in endothelial basal medium‐2 (EBM‐2; Lonza) supplemented with endothelial growth medium‐2 (EGM‐2) additives. Human dermal fibroblasts (Cat. No: CCD‐986sk) were obtained from the Korean Cell Line Bank (KCLB) and cultured in DMEM with 10% FBS. In parallel, human macrophages (Cat. No.: CRL‐9855, ATCC) were cultured in RPMI‐1640 medium supplemented with 10% FBS and 1% penicillin/streptomycin. Elongated and spindle‐shaped human myometrial cells were isolated lower segment of myometrial biopsies. Similar to the isolation process of endometrial stem cells, myometrial samples were mechanically minced into small pieces, and then the small pieces were enzymatically digested in DMEM containing 10% FBS and 250 U mL^−1^ type I collagenase under continuous shaking at 37 °C for 5 h. The digested solutions were filtered through a mesh size 70 µm cell strainer to remove leftover undigested tissue fragments then filtered again through a mesh size 40 µm cell strainer to separate spindle‐shaped human myometrial cell populations from epithelial gland cells and their aggregates. Given the intrinsic proliferation limits of primary human cells during extended in vitro culture,^[^ ^55^ ^]^ all cell types were subjected to stable immortalization through lentiviral transduction of the SV40 large T antigen gene. This approach has been shown to maintain the core phenotypic characteristics of parental cells while enabling long‐term propagation.^[^ ^56^ ^]^ While this study incorporated SV40 large T antigen–immortalized macrophages and endothelial cells to ensure stable, long‐term culture and reproducibility within the microfluidic system, we acknowledge that immortalization may alter certain cellular pathways and toxicant responses compared to primary cells. However, primary cells present their own limitations, including inter‐donor variability, limited proliferative capacity, and inconsistent performance across replicates. In contrast, the use of immortalized lines enabled consistent biomarker induction and assay repeatability, which are essential for establishing a reliable toxicity screening platform. Thus, while accepting the trade‐offs, we prioritized the practical advantages of using standardized cell lines at this stage of development.

### Isolation and Immortalization of Human Ovarian Granulosa and Theca Cells

Human ovarian granulosa and theca cells were isolated from follicular aspirates collected during oocyte retrieval procedures from in vitro fertilization (IVF) patients. All participants provided written informed consent, and protocols were approved by the Institutional Review Board of Gachon University (IRB No: GAIRB2019‐316). Patient exclusion criteria: Patients diagnosed with polycystic ovary syndrome (PCOS), premature ovarian insufficiency (POI), endometriosis, ovarian tumors, or systemic inflammatory/autoimmune disorders were excluded from cell collection to ensure the use of physiologically normal ovarian tissue. The isolation procedure was adapted from previously reported methodologies with minor modification.^[^ ^32^ ^]^ Following centrifugation of the follicular aspirates at 500 × g for 5 min, the cell pellet was resuspended in a 3 mL mixture of M‐199 and MCDB‐105 (1:1) supplemented with 10% fetal bovine serum (FBS). Granulosa and theca cells were then enriched via centrifugation on a single‐layer Percoll gradient at 2800 × g for 20 min. Due to the inherently limited proliferative capacity of primary granulosa and theca cells, which generally restricts their culture viability to only a few days,^[^ ^57^ ^]^ cells were stably transduced with the SV40 large T antigen to promote extended propagation while preserving key phenotypic characteristics.^[^ ^56^ ^]^ Immortalized granulosa and theca cell lines were clonally expanded to ensure population homogeneity and minimize variability often encountered in primary cell cultures. Following immortalization, both cell types were maintained in M‐199/MCDB‐105 (1:1) medium supplemented with 10% FBS at 37 °C in a humidified incubator with 5% CO_2_. The medium was refreshed every 48 h to maintain optimal culture conditions.

### Fabrication of the Organ‐on‐a‐Chip Device via 3D Printed Casting Molds

To construct the organ‐on‐a‐chip platform simulating ovarian and endometrial architecture with cyclic dynamics, a custom‐designed casting mold was fabricated using a 3D printing process. First, the anatomical layout of the ovary and uterine endometrium was digitally modeled using computer‐aided design (CAD) software (Figure 1B). The completed 3D CAD model was converted into surface tessellation language (STL) files, which represent the geometry through a series of vector‐based surface meshes. These STL data were then digitally sliced into 50 µm‐thick layers along the Z‐axis and processed using a digital light processing (DLP) 3D printer (Master EV, Carima, Seoul, Korea) to create a high‐resolution casting mold from poly(lactic acid) (PLA) via a layer‐by‐layer photopolymerization approach. Subsequently, polydimethylsiloxane (PDMS; Sylgard 184, Dow Corning, MI, USA) was prepared by mixing the elastomer base and curing agent at a 20:3 (w/w) ratio. The mixture was vigorously blended for 10 min at ambient temperature to ensure uniform dispersion of the curing components. The homogenized PDMS solution was then carefully poured into the PLA casting mold and degassed in a vacuum desiccator to eliminate entrapped air bubbles. Polymerization was performed in a convection oven at 65 °C for 24 h. Upon complete curing, the mold was cooled to room temperature, and the solidified PDMS chip was gently demolded using a precision razor blade to avoid structural deformation or damage to the microfluidic features (Figure 1C). To recapitulate the native extracellular matrix (ECM) microenvironment of ovarian and endometrial tissues within the chip chambers, a composite hydrogel was formulated using collagen, hyaluronic acid, fibrinogen, and thrombin. The concentrations of collagen (3 mg mL^−1^), hyaluronic acid (0.5 mg mL^−1^), fibrinogen (10 mg mL^−1^), and thrombin (2 U mL^−1^) were selected based on preliminary optimization experiments conducted in our laboratory, targeting optimal matrix stiffness, porosity, and cell viability for ovarian and endometrial cells in 3D culture. Optimization results were further supported by previously published protocols for reproductive tissue engineering.^[^ ^34^ ^]^ Lower collagen concentrations (<1.5 mg mL^−1^) led to inadequate structural stability and excessive gel contraction over time, while higher concentrations (>4 mg mL^−1^) negatively affected cell proliferation and viability due to increased stiffness. The inclusion of hyaluronic acid enhanced matrix hydration and elasticity, mimicking the viscoelastic characteristics of the native reproductive tract. Fibrinogen and thrombin were incorporated to promote rapid gel crosslinking and structural stabilization without compromising nutrient diffusion or cell viability. This composite hydrogel was prepared fresh prior to cell encapsulation and loaded into the PDMS chip chambers to support long‐term 3D culture under microfluidic conditions.

### Encapsulating Various Types of Ovarian and Uterine Endometrial Cells within the Multi‐Compartment Organ‐on‐a‐Chip Platform

Various ovarian and endometrial cell types were embedded in distinct compartments of the PDMS‐based organ‐on‐a‐chip device using a multi‐component natural hydrogel system. For this purpose, type I collagen (3 mg mL^−1^; Sigma‐Aldrich, C5483) and hyaluronic acid (3 mg mL^−1^; Sigma‐Aldrich, 935166) were dissolved in DMEM and prepared as base hydrogel solutions. To enhance gel stability and mechanical integrity, fibrinogen (12.5 mg mL^−1^; Lee Biosolutions, 528–50) and thrombin (1.25 U mL^−1^; Sigma‐Aldrich, F3879‐5G) were additionally solubilized in DMEM and combined with the collagen‐hyaluronic acid matrix. A unified culture medium compatible with multiple reproductive cell types was formulated using DMEM supplemented with 15 mM HEPES, 200 µg mL^−1^ BSA, 2.5 ng mL^−1^ EGF, 0.01 mM non‐essential amino acids, 5% FBS, and 1% penicillin/streptomycin. Equal volumes of collagen, hyaluronic acid, fibrinogen, and thrombin solutions were mixed in a 1:1:1:1 ratio and used as the embedding matrix for the target cells. Human primary cells—including theca cells, granulosa cells, endometrial stromal stem cells, HUVECs, and uterine macrophages—were suspended at a final density of ≈1 × 10⁶ cells mL^−1^ in the composite hydrogel solution. Each cell‐laden hydrogel mixture was loaded into its corresponding compartment of the pre‐fabricated organ‐on‐a‐chip device using a sterile syringe. The loaded constructs were incubated at ambient temperature for 30 min to facilitate in situ gelation. To support sustained viability and phenotypic maintenance, each compartment was supplied with 500 µL of a mixed culture medium composed of DMEM, EGM‐2, and EMEM in a 1:1:1 volumetric ratio supplemented with 5% FBS. Culture media were replaced every two days under static incubation conditions.

### Mechanical Characterization of Bioengineered Reproductive Tissue Constructs: Microstructural Morphology, Compressive Mechanics, and Rheological Behavior

Microstructural Analysis: To evaluate the internal architecture of hydrogel‐based constructs, samples were freeze‐dried and coated with a 10 nm gold/palladium layer via ion sputtering (15 mA, 30 s; Hitachi Ion Sputter 1010). SEM imaging (EVO LS10, Carl Zeiss) was performed at 1.2–1.3 kV at the Korean Basic Science Institute (KBSI), capturing both transverse and longitudinal sections to account for structural heterogeneity.^[^ ^58^ ^]^ Compressive Mechanical Testing: Disc‐shaped samples (10 mm diameter, 3 mm height) were subjected to uniaxial compression using a universal testing machine (QM100S, QMESYS, Korea) at a rate of 5 mm/min until failure. Stress–strain curves were analyzed to determine the elastic modulus and ultimate compressive strength, following established mechanical protocols.^[^ ^58^ ^]^ Rheological Assessment: Viscosity and flow characteristics were assessed at 37 °C using a rotational rheometer (MCR 102, Anton Paar, Switzerland). Shear rates ranging from 1 to 20 s^−1^ were applied to examine non‐Newtonian behaviors, including shear thinning or thickening. All measurements were conducted under isothermal conditions, and data were analyzed in line with previously reported methods.^[^ ^58^ ^]^

### Assessment of Long‐Term Cell Viability within the Organ‐on‐a‐Chip System

To monitor the sustained viability of encapsulated cells within each compartment of the engineered organ‐on‐a‐chip platform, a fluorescence‐based viability assay was conducted at defined intervals (Days 1, 7, 14, 21, and 28 post‐embedding) using the Live/Dead Viability/Cytotoxicity Kit (Invitrogen, L3224). Prior to staining, chip compartments were gently washed three times with serum‐free DMEM to eliminate residual serum components. Subsequently, 1 mL of staining solution—comprising 2 µM ethidium homodimer‐1 and 4 µM calcein AM in PBS—was introduced into each compartment and incubated for 30 min at ambient temperature. Fluorescent signals corresponding to viable (green, calcein+) and non‐viable (red, ethidium+) cells were captured using the EVOS FL Cell Imaging System (Thermo Fisher Scientific, USA). This approach enabled spatial and temporal evaluation of cell survival across the biomimetic compartments of the platform under static culture conditions.

### Quantitative Evaluation of Sustained Metabolic Activity in Organ‐on‐a‐Chip Compartments

To assess long‐term metabolic activity of cells embedded within distinct compartments of the organ‐on‐a‐chip platform, a colorimetric assay based on water‐soluble tetrazolium salt (CCK‐8; Abbkine, Cat. No. KTC011001) was employed following the manufacturer's protocol. After culture within the chip, the hydrogel‐based ovarian and endometrial tissue constructs containing the embedded cells were carefully retrieved from the PDMS chip using sterile dissection tools. These excised constructs, each standardized to a consistent size and volume, were then transferred into individual wells of a 96‐well plate, effectively converting the sample format into a conventional assay‐compatible configuration. Once transferred, the CCK‐8 assay was performed according to the standard manufacturer's protocol, as cells within the hydrogel constructs were sufficiently exposed to the assay reagent. Briefly, 100 µL of CCK‐8 reagent diluted in serum‐free DMEM was added to each compartment of the device, and the chips were incubated for 4 h at 37 °C under 5% CO_2_. Cellular metabolic activity was subsequently quantified by measuring the absorbance at 450 nm using a microplate spectrophotometer (SoftMax Pro 5, Molecular Devices, San Jose, CA, USA). This analysis enabled comparative evaluation of cell viability and metabolic function across tissue‐specific compartments over extended culture durations.

### Immunofluorescence Staining of Various Cell Types Embedded in the Organ‐on‐a‐Chip

For antibody‐based fluorescence imaging, the organ‐on‐a‐chip constructs were initially fixed with 4% paraformaldehyde, followed by permeabilization using a solution containing 0.4 M glycine and 0.3% Triton X‐100. To minimize nonspecific antibody binding, samples were incubated with 2% normal swine serum (DAKO, Glostrup, Denmark) as a blocking agent. Immunostaining was subsequently conducted using a panel of primary antibodies targeting specific cellular markers and reporter proteins, including anti‐mCherry (TAKARA, Cat. No.: 632524), anti‐GFP (Invitrogen, Cat. No.: V820‐20), FSH receptor (NOVUS, Cat. No.: NBP2‐36489), LH receptor (Abcam, Cat. No.: ab125214), estrogen receptor (Santa Cruz Biotechnology, Cat. No.: sc‐8005), progesterone receptor (Santa Cruz Biotechnology, Cat. No.: sc‐810), CK14 (Santa Cruz Biotechnology, Cat. No.: sc‐58951), MyoD (Santa Cruz Biotechnology, Cat. No.: sc‐377460), Myogenin (Abcam, Cat. No.: ab1835), CD11b (Abcam, Cat. No.: ab52478), CD68 (Abcam, Cat. No.: ab125212), and Prostaglandin E2 (Abcam, Cat. No.: ab167171), CD31 (R&D Systems, Cat. No.: BBA7), vimentin (BD Biosciences, Cat. No.: 550513), and von Willebrand factor (vWF; Abcam, Cat. No.: ab6994). Fluorescence imaging was performed using a confocal laser scanning microscope (Zeiss LSM 510 Meta) to visualize marker‐specific expression patterns and validate the identity of the compartmentalized cell populations.

### Protein Isolation and Western Blot Analysis

Total cellular proteins were extracted using a lysis buffer composed of 50 mM Tris‐HCl (pH 7.5), 150 mM NaCl, 5 mM EDTA, 1 mM dithiothreitol (DTT), 0.01% NP‐40, and 0.2 mM phenylmethylsulfonyl fluoride (PMSF). Protein concentrations were quantified using a BSA‐based standard curve via a Bradford assay. Equal amounts of protein (normalized by concentration) from each lysate were subjected to SDS–polyacrylamide gel electrophoresis (SDS–PAGE) and subsequently transferred to nitrocellulose membranes (Bio‐Rad, Hercules, CA, USA) using a semi‐dry blotting system. Membranes were blocked for 1 h at room temperature in Tris‐buffered saline containing 0.1% Tween‐20 (TBS‐T) supplemented with 5% non‐fat dry milk to reduce nonspecific binding. Primary antibody incubations were carried out overnight at 4 °C using the following antibodies: anti‐ANGPTL4 (Santa Cruz, sc‐373762), anti‐SERPINB2 (Abcam, ab47742), anti‐MMP‐2 (Cell Signaling Technology, #4022), anti‐MMP‐9 (CST, #13667), anti‐caspase‐3 (CST, #9662), and anti‐β‐actin (Abcam, ab189073) as a loading control. Following extensive washing with TBS‐T, membranes were incubated for 1 h at room temperature with horseradish peroxidase (HRP)‐conjugated secondary antibodies—goat anti‐rabbit IgG (BD Pharmingen, 554021) or goat anti‐mouse IgG (BD Pharmingen, 554002). Protein bands were visualized using an enhanced chemiluminescence (ECL) detection system, and signal intensity was analyzed using image processing software where applicable.

### Transwell‐Based Cell Migration and Invasion Assay

Cell motility was assessed using a Transwell system (Corning, NY, USA) equipped with polycarbonate membranes (diameter: 6.5 mm; pore size: 8.0 µm) fitted in a 24‐well format. A total of 1 × 10⁵ cells in serum‐free medium were seeded into the upper chambers, while the lower wells were filled with complete medium containing 10% FBS to serve as a chemoattractant. After incubation for 24 h at 37 °C under standard culture conditions, non‐migrated cells were carefully removed from the upper surface of the membrane using sterile cotton‐tipped swabs. Cells that traversed the membrane and adhered to the underside were fixed with 4% paraformaldehyde for 5 min, followed by staining with hematoxylin for 15 min. The number of migrated or invaded cells was quantified under a light microscope at 50× magnification by counting three randomly selected microscopic fields per membrane. All assays were performed in triplicate, and results were expressed as mean cell number ± standard deviation.

### shRNA‐Mediated Knockdown of ANGPTL4 and SERPINB2

To selectively suppress ANGPTL4 and SERPINB2 expression, gene‐specific short hairpin RNAs (shRNAs) targeting human ANGPTL4 (Accession No. NM_139314) and SERPINB2 (Accession No. NM_002575), along with a scrambled non‐targeting control shRNA, were procured from Bioneer (Daejeon, South Korea). Transfections were carried out using Lipofectamine 2000 (Invitrogen, Cat. No. 52887) following the manufacturer's protocol. Briefly, 3 µg of shRNA plasmid and 3 µL of Lipofectamine 2000 were separately diluted in serum‐ and antibiotic‐free Opti‐MEM medium (Gibco), combined, and incubated at room temperature for 20 min to allow complex formation. Endometrial stem cells were seeded in 6‐well plates and incubated in Opti‐MEM for 5 h prior to transfection. The shRNA–Lipofectamine complexes were then added to the cells. Five hours post‐transfection, the medium was replaced with StemPro MSC SFM CTS (Thermo Fisher Scientific) supplemented with 10% FBS to facilitate recovery and continued culture. Transfection efficiency was verified by quantitative PCR and/or immunoblotting to confirm effective mRNA knockdown.

### Quantification of Hormonal Secretion via ELISA

To assess hormone secretion profiles of compartmentalized ovarian and endometrial cells within the organ‐on‐a‐chip system, concentrations of estradiol (Abnova Corporation, Cat. No.: KA3394), progesterone (Abnova Corporation, Cat. No.: KA0299), and PGE2 (Abnova Corporation, Cat. No.: KA0326) were measured using enzyme‐linked immunosorbent assay (ELISA) kits. All procedures were performed following the manufacturer's instructions. Conditioned media were collected at defined time points and centrifuged at 300 × g for 5 min to remove residual debris. Subsequently, 100 µL of each supernatant or standard solution was added to designated wells of the pre‐coated 96‐well ELISA plates. After incubation with hormone‐specific detection antibodies and colorimetric substrates, absorbance was recorded at 450 nm using a calibrated microplate reader (SpectraMax iD3, Molecular Devices, USA).

### Statistical Analysis

All quantitative data were statistically analyzed using GraphPad Prism version 9.0 (GraphPad Software, San Diego, CA, USA). For comparisons between two experimental groups, unpaired two‐tailed Student's t‐tests were employed under the assumption of equal variance. Data are presented as mean ± standard deviation (SD), unless otherwise specified. A p‐value less than 0.05 was considered statistically significant. All experiments were conducted with at least three independent biological replicates to ensure analytical robustness.

## Conflict of Interest

The authors declare no conflict of interest.

## Author Contributions

S.R.K., E.K.M., C.M.L. contributed equally to this work. S.R.K., E.K.M., C.M.L., C.H.P., B.C.O., and Y.J.J. were responsible for experiment design and execution, data analysis, and manuscript preparation. C.H.P., B.C.O., Y.J.J., and I.S.H. contributed to experimental design, data analysis, and manuscript writing.

## Supporting information



Supporting Information

## Data Availability

The data that support the findings of this study are available from the corresponding author upon reasonable request.
