# Supplementary material for: A Biomarker‐Driven Ovary–Endometrium Organ‐on‐a‐Chip Mimicking 3D Multicellular Complexity and Menstrual Cyclicity for Predicting Reproductive Toxicity
Source: Adv Sci (Weinh). 2026 Jan 15;13(30):e11098. doi: 10.1002/advs.202511098 (PMC13248849; doi:10.1002/advs.202511098)
Supplement: Supplementary file 1 — Supporting Information [file ADVS-13-e11098-s001.pdf]

Supplementary information

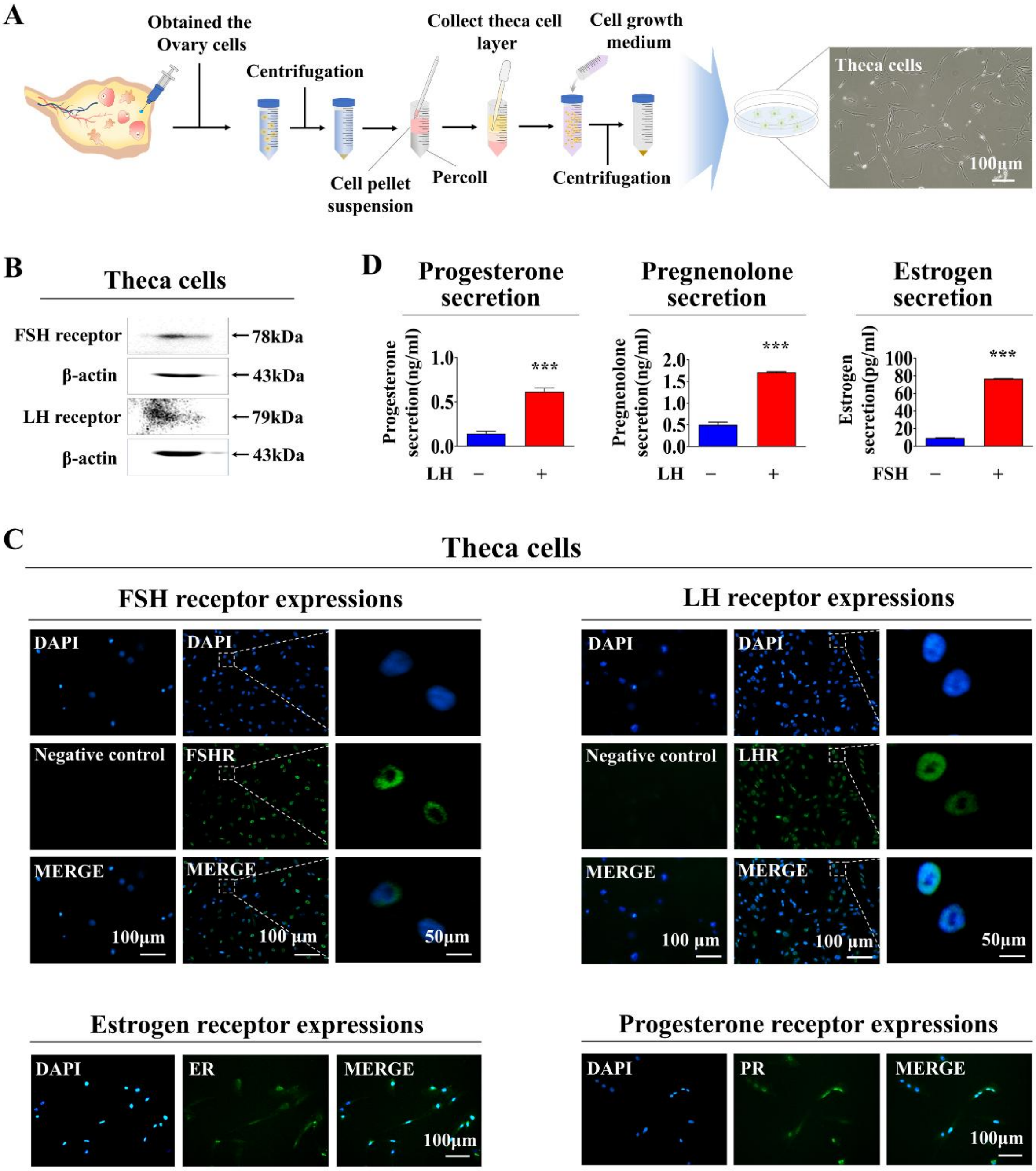

Figure S1. Isolation and characterization of human theca cells from IVF- associated follicular aspirates. A schematic representation outlines the isolation method for human theca cells. Follicular aspirates underwent density gradient centrifugation with Percoll, after which the theca cell layers were selectively harvested, washed, and cultured in a suitable growth medium **(A)**. Western blotting was conducted to assess gonadotropin receptor expression in isolated theca cells. Robust expression of

follicle-stimulating hormone receptor (FSHR, 79 kDa) and luteinizing hormone receptor (LHR, 78 kDa) was observed in the isolated population, confirming their follicular origin and specific receptor profiles. β-actin served as a loading control **(B)**. Immunofluorescence analysis demonstrated strong immunoreactivity for FSHR and LHR (green), as well as marked nuclear localization of estrogen receptor (ER) and progesterone receptor (PR), collectively validating the endocrine function and follicular identity of the isolated ovarian cells **(C)**. ELISA quantification of steroid hormone secretion following gonadotropin treatment revealed that LH markedly increased progesterone and pregnenolone output, while FSH enhanced estrogen secretion **(D)**. DAPI staining (blue) marks the nuclei. Scale bars, 100 μm. β-actin was used as the internal control. Data are presented as mean ± SEM; $^{*}P < 0.05$, $^{**}P < 0.01$, $^{***}P < 0.001$.

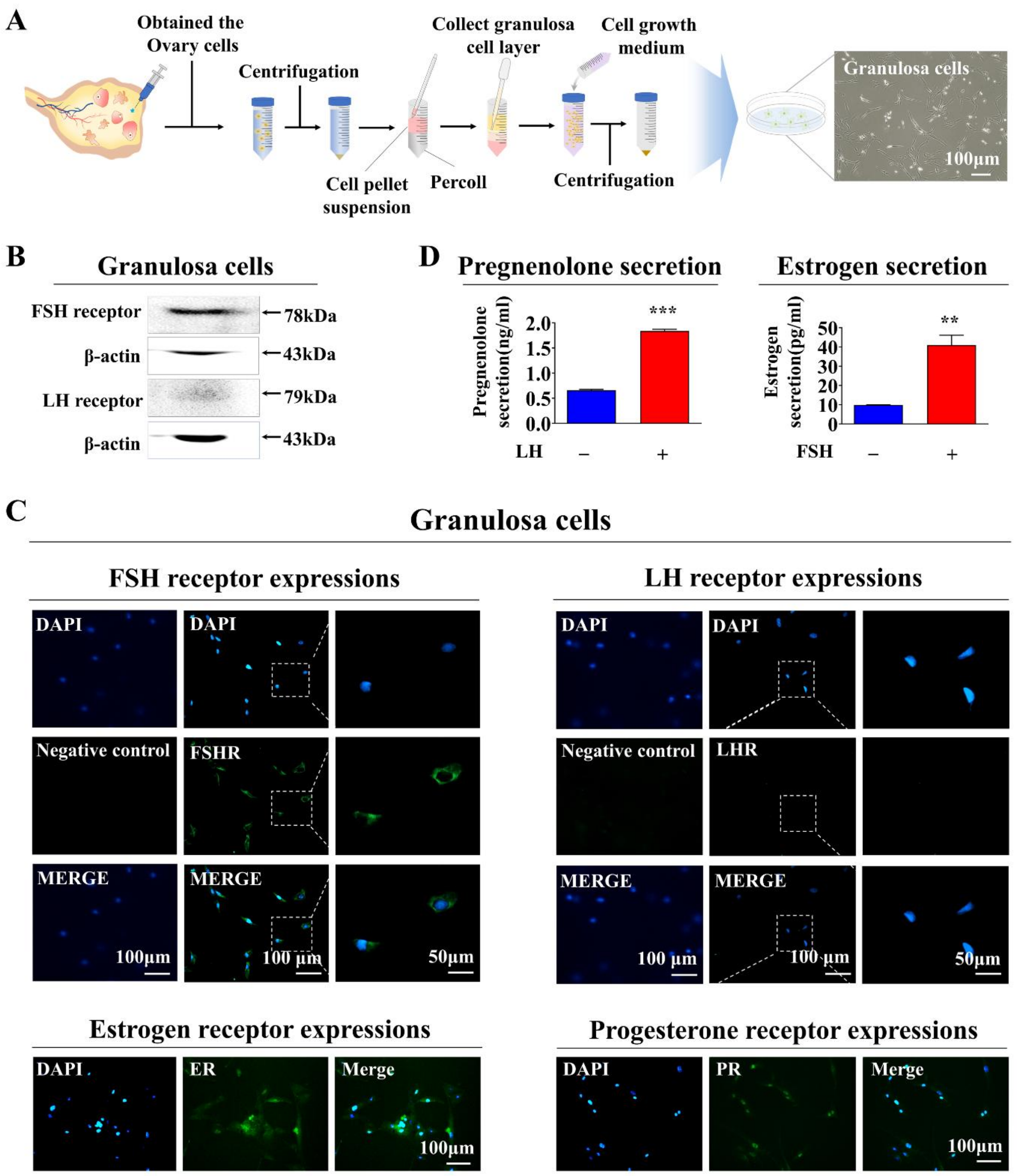

Figure S2. Isolation and characterization of human granulosa cells from IVF- associated follicular aspirates. This schematic illustrates the process of isolating granulosa cells from IVF- associated follicular aspirates. After separation by Percoll density gradient centrifugation, granulosa cell layers were collected and maintained in standard culture medium **(A)**. The isolated granulosa cells displayed high expression of follicle-stimulating hormone receptor (FSHR, 79 kDa) and lower levels of

luteinizing hormone receptor (LHR, 78 kDa), confirming their follicular origin and stage-specific molecular profile **(B)**. Immunofluorescence staining revealed robust FSHR and relatively weaker LHR immunoreactivity (green), alongside clear nuclear localization of estrogen receptor (ER) and progesterone receptor (PR), supporting the endocrine competence and follicular lineage characteristics of the isolated cells **(C)**. ELISA was used to quantify hormone secretion from granulosa cells after gonadotropin exposure. LH treatment notably enhanced pregnenolone production, and FSH stimulation led to increased estrogen output **(D)**. DAPI staining (blue) identifies nuclei. Scale bars, 100 μm. β-actin was used as the internal control. Data are presented as mean ± SEM; *$P < 0.05$, **$P < 0.01$, ***$P < 0.001$.

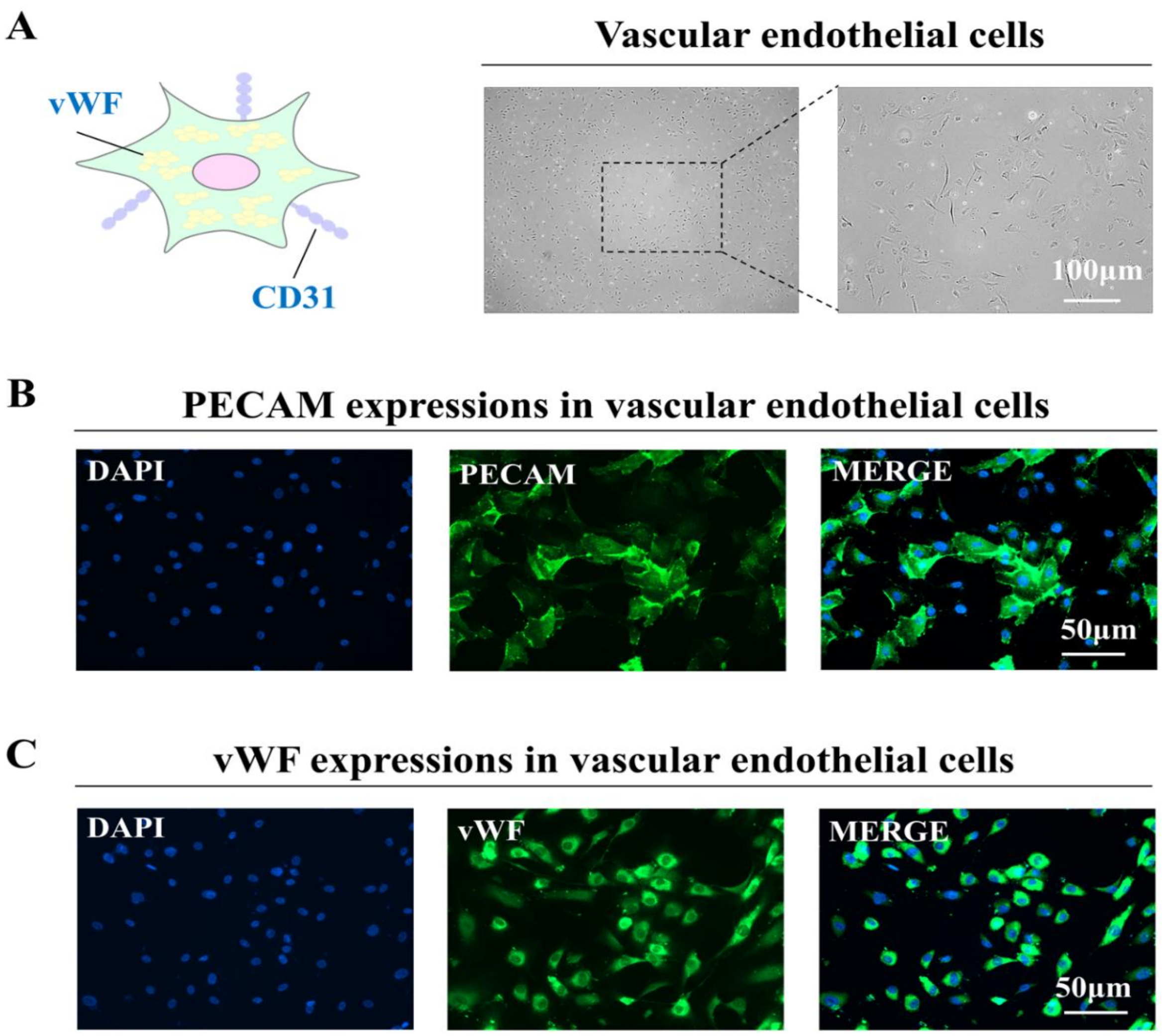

Figure S3. Phenotypic characterization of vascular endothelial cells for application in the organ on a chip platform. Phase-contrast microscopy demonstrates that cultured human umbilical vein endothelial cells (HUVECs) assume a typical cobblestone-like appearance, indicative of a confluent endothelial monolayer **(A)**. Immunofluorescence analysis shows robust PECAM-1 (CD31; green) signal concentrated at cell junctions, with nuclear counterstaining by DAPI (blue), thereby confirming endothelial phenotype and barrier-forming capacity **(B)**. Immunofluorescent detection of von Willebrand factor (vWF; green) displays a distinct intracellular punctate pattern, reflecting endothelial identity and secretory granule assembly **(C)**. Nuclei are counterstained with DAPI (blue). Scale bars: 100 µm (A); 50 µm (B–C).

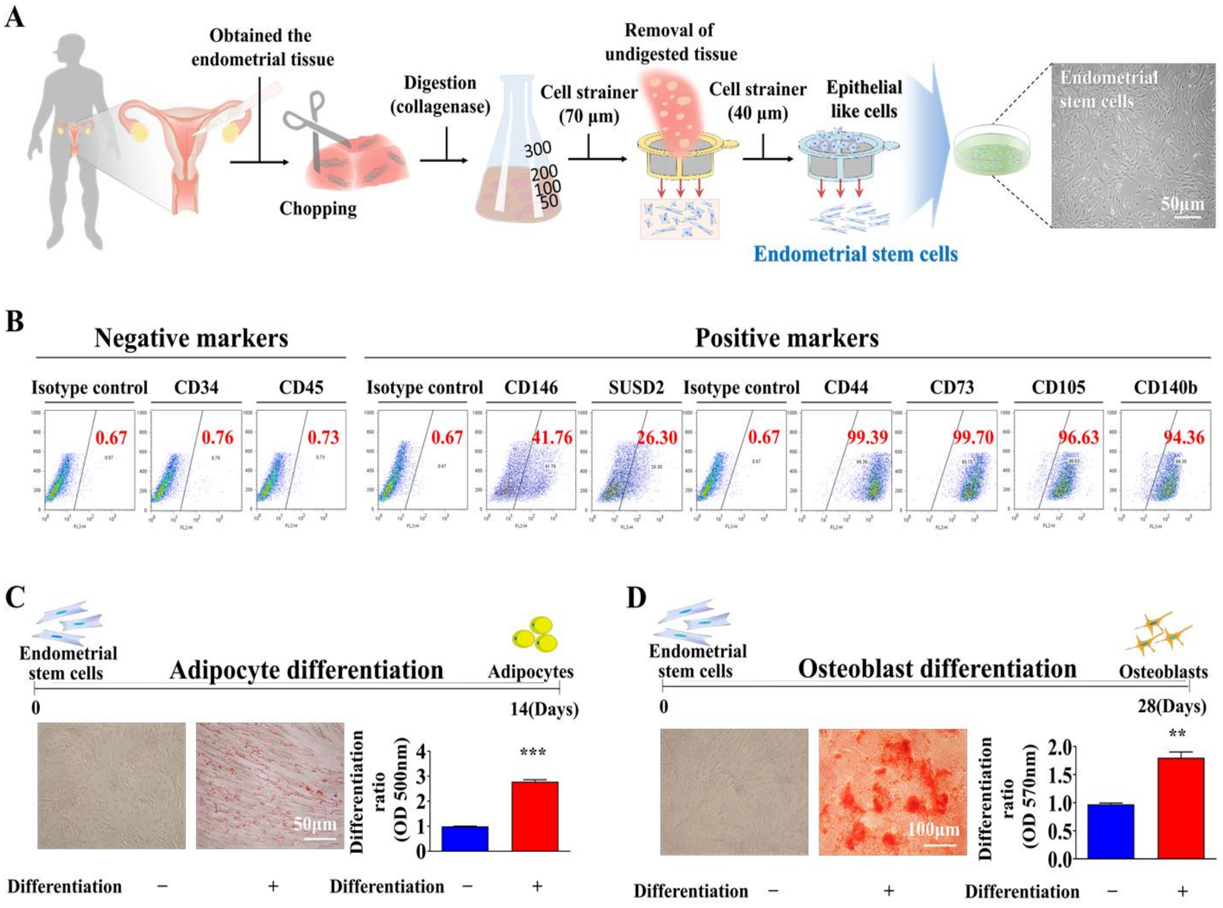

Figure S4. Isolation, phenotypic characterization, and multipotent differentiation of human endometrial stem cells. Illustration of a collagenase-based enzymatic digestion strategy for isolating human endometrial stem cells from biopsy-derived tissue samples. After mechanical mincing and enzymatic digestion, the resultant cell suspension was sequentially filtered using 70 µm and 40 µm strainers to efficiently deplete epithelial-like cells and undigested tissue fragments, thereby enriching the stem cell population **(A)**. Flow cytometry of isolated endometrial stem cells confirmed the presence of established mesenchymal stem cell markers (CD44, CD73, CD105, CD140b, CD146, and W5C5/SUSD2), with minimal detection of hematopoietic lineage markers (CD34 and CD45), supporting their mesenchymal identity and stemness **(B)**. Adipogenic differentiation was evidenced by Oil Red O staining, which detected intracellular lipid droplet formation. Quantitative assessment demonstrated pronounced adipogenic induction, as measured by OD at 500 nm **(C)**. Osteogenic differentiation was verified using Alizarin Red S staining, which identified extracellular calcium deposits. Quantification of stain intensity indicated significant osteogenesis, confirmed by OD readings

at 570 nm (D). Scale bars: 100 μm; 50 μm. Data are expressed as mean ± SEM; $^{*}P < 0.05$, $^{**}P < 0.01$, $^{***}P < 0.001$.

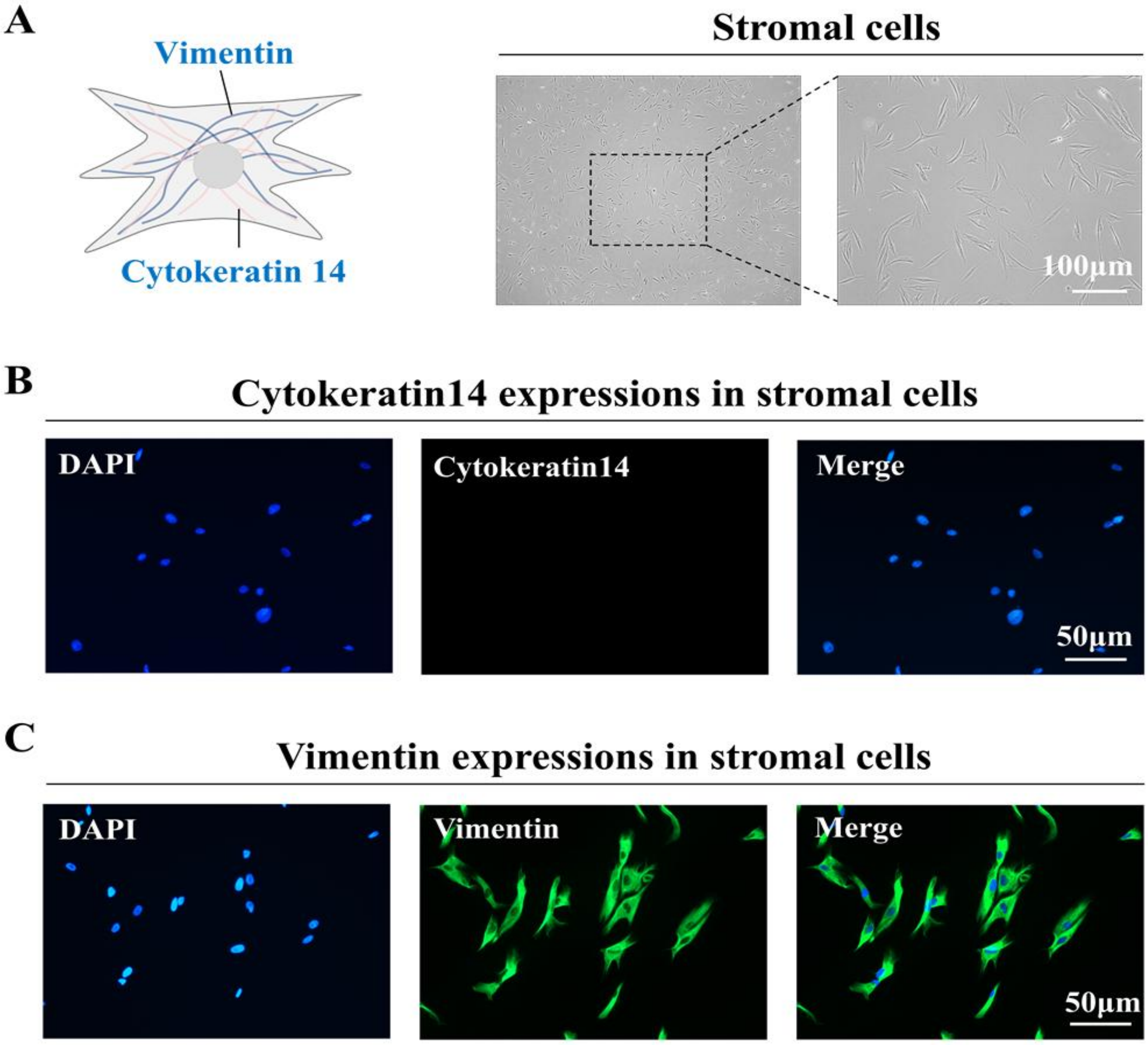

Figure S5. Characterization of human fibroblasts as a surrogate model for endometrial stromal cells. Phase-contrast microscopy demonstrates that cultured human fibroblasts possess a spindle-shaped morphology, which is indicative of their mesenchymal origin and is frequently seen in stromal cell populations **(A)**. The lack of cytokeratin 14 (CK14, green) expression further substantiates the non-epithelial, fibroblast-like nature of these cells **(B)**. Immunocytochemical staining reveals strong vimentin (green) expression, an established mesenchymal marker, thereby confirming the stromal phenotype of the fibroblasts **(C)**. Collectively, these findings indicate that human fibroblasts maintain a stromal-like phenotype and are suitable for use as a model for endometrial stromal cells in the organ-on-a-chip system. Scale bars: 50 μm.

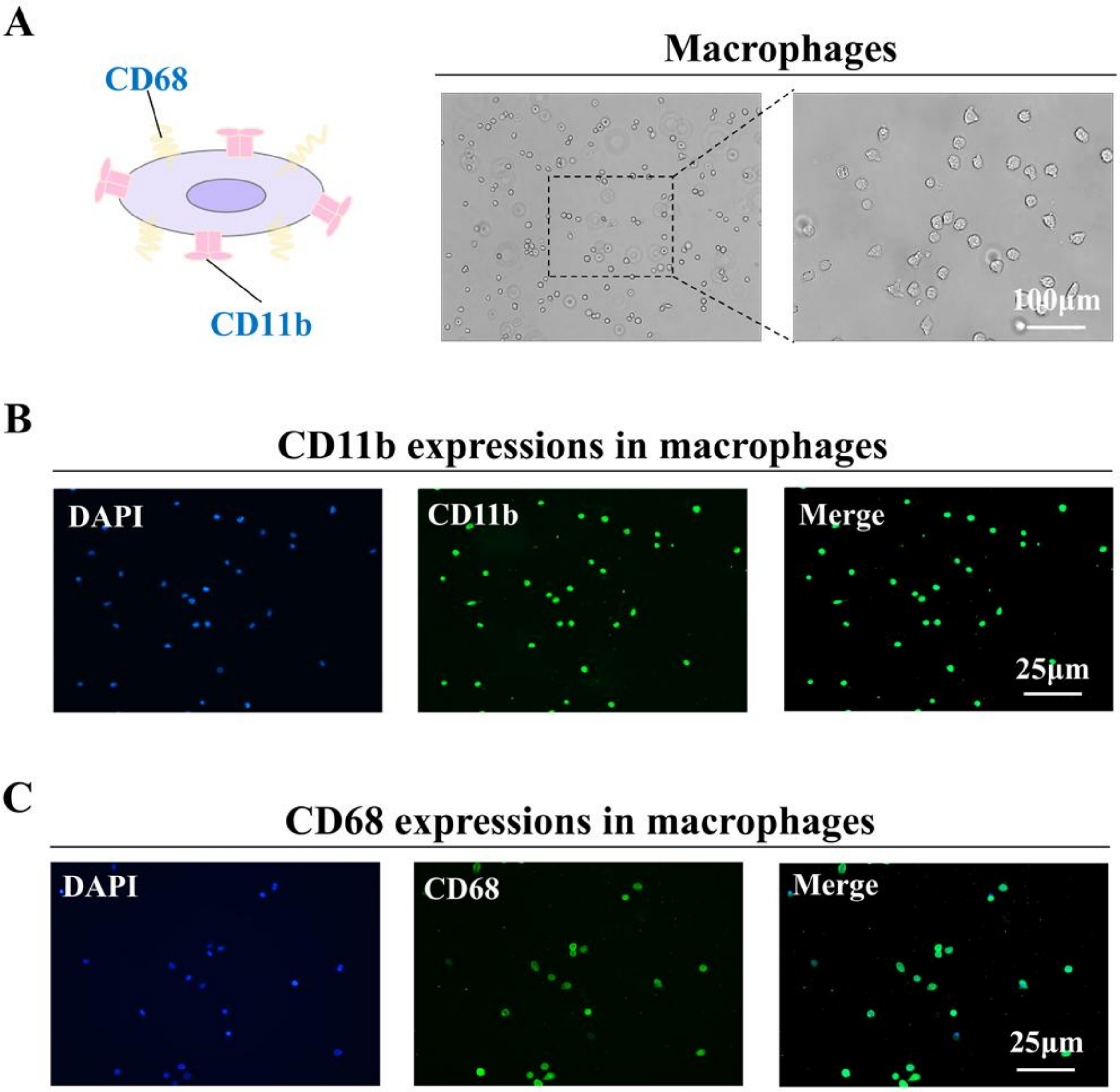

Figure S6. Characterization of human macrophages as immune components within the organ-on-a-chip platform. Phase-contrast microscopy confirms that cultured human macrophages display a typical round morphology, consistent with their classification as tissue-resident immune cells. Immunofluorescence analysis shows strong CD11b **(B)** and CD68 **(C)** immunoreactivity, confirming both the myeloid lineage and the activation status of these macrophages. Scale bars = 100 μm; 25 μm.

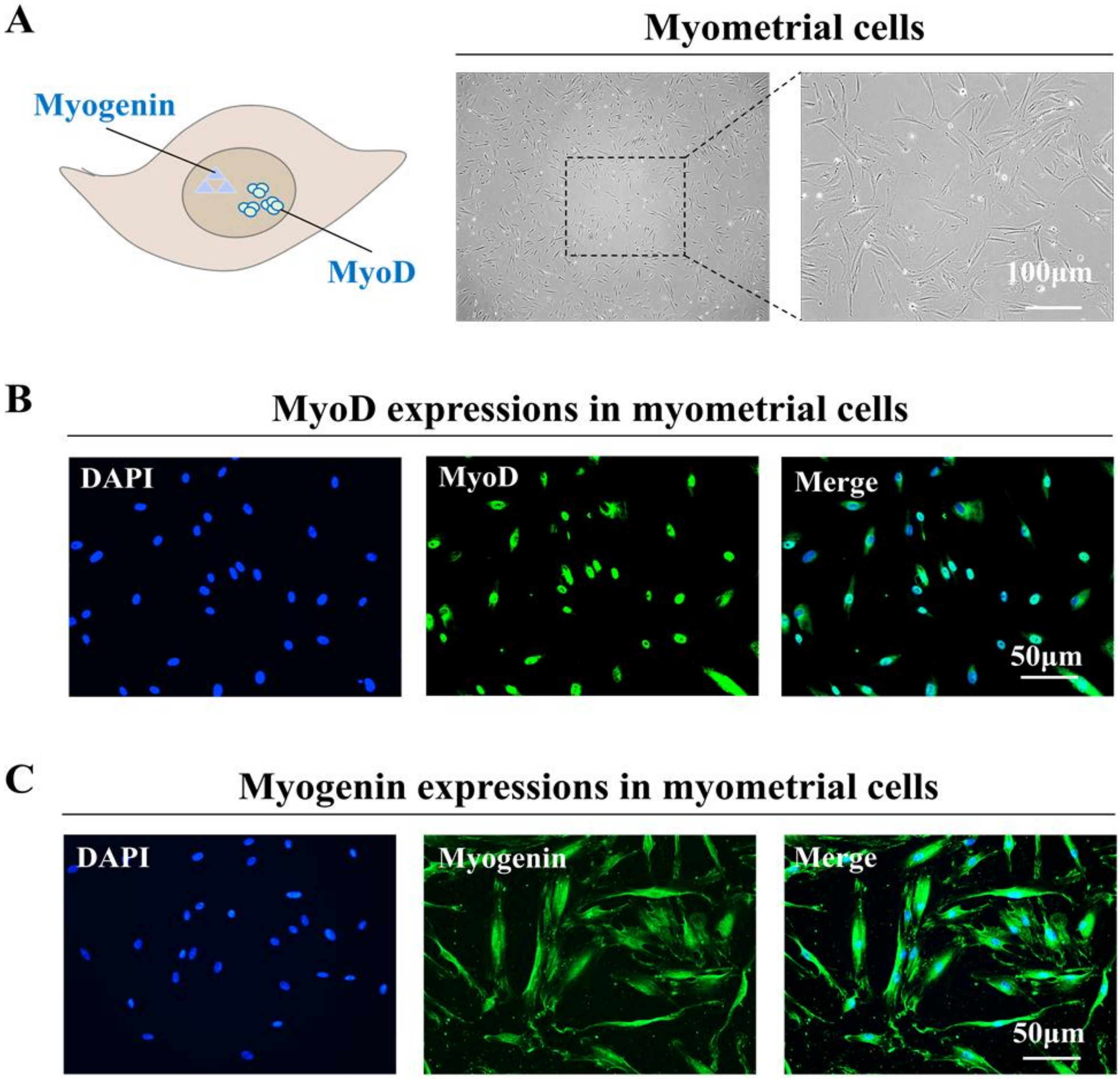

Figure S7. Characterization of human myometrial cells incorporated into the endometrial muscle compartment. Phase-contrast imaging shows that human myometrial cells display the expected elongated, spindle-shaped morphology indicative of their smooth muscle lineage **(A)**. Immunofluorescence staining validates the presence of MyoD (green), a key myogenic regulatory factor involved in muscle cell differentiation and specification **(B)**. Further immunostaining for myogenin (green), a vital muscle-specific transcription factor, supports the identity of the cultured cells as being of myometrial origin **(C)**. DAPI was utilized for nuclear counterstaining (blue). Scale bars: 1000 μm (A); 50 μm (B, C).

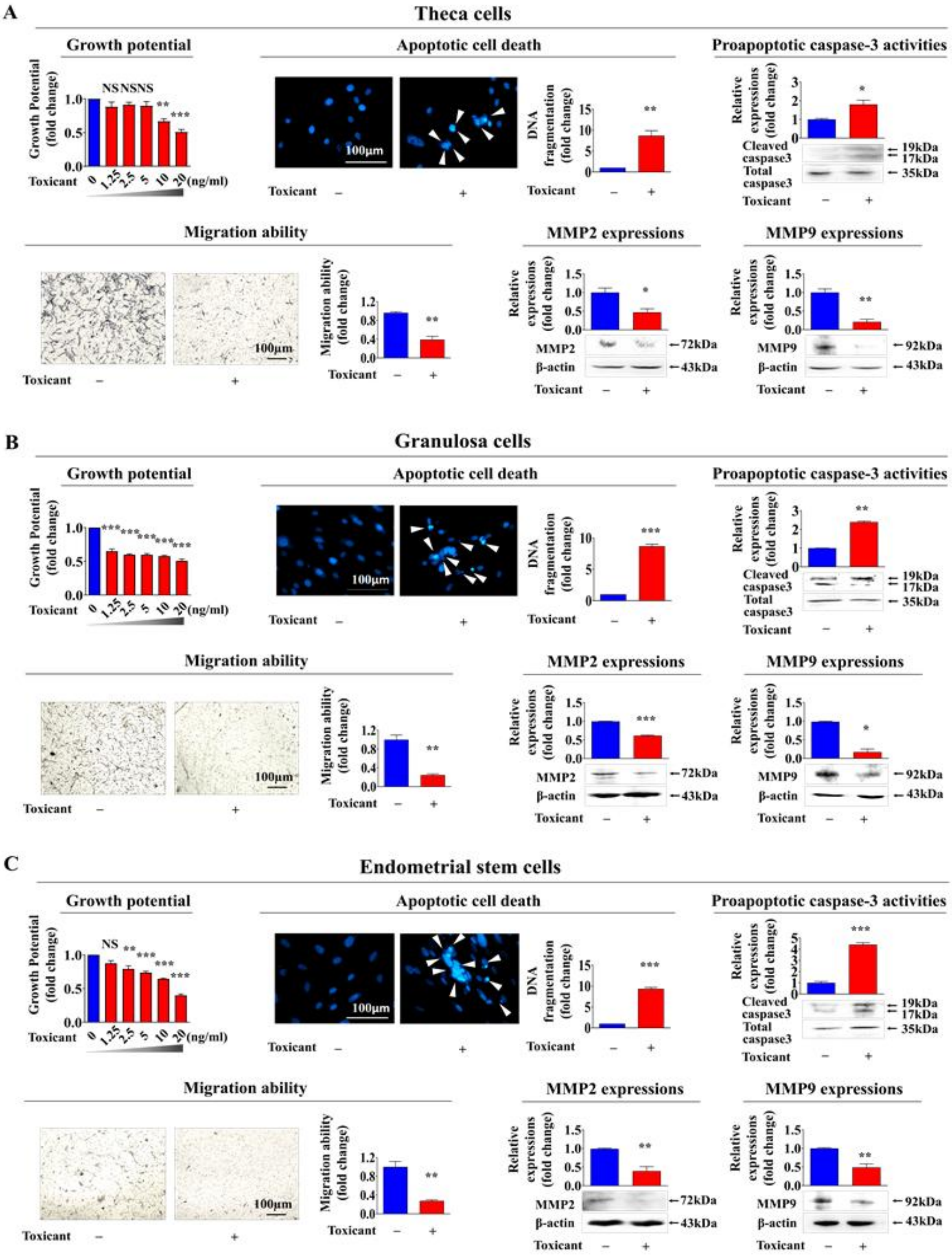

Figure S8. Dose-dependent evaluation of standard toxicant-induced toxicity in major female reproductive cell types. Toxicity profiling of theca cells*:* exposure to increasing concentrations of standard toxicant (dioxin) significantly diminished cellular proliferation and intensified apoptotic

responses, as demonstrated by DNA fragmentation assays and elevated levels of cleaved caspase-3. In addition, standard toxicant reduced the migratory capacity as well as the expression of matrix metalloproteinase-2 (MMP-2) and MMP-9, evaluated through Transwell migration assays and western blot analyses, respectively **(A)**. Toxicological response of granulosa cells: toxicant exposure led to a concentration-dependent decline in cell growth and a corresponding elevation in apoptosis and caspase-3 activation. Furthermore, granulosa cells displayed diminished migration and reduced MMP-2 and MMP-9 levels, supporting the conclusion that toxicant exposure triggers extracellular matrix remodeling alongside cytotoxic effects **(B)**. Assessment of toxicity in endometrial stem cells: following toxicant treatment, endometrial stem cells exhibited pronounced reductions in proliferation with concomitant elevations in apoptosis, as indicated by DNA fragmentation and caspase-3 cleavage. Consistently, cell migration was substantially impaired and there was notable downregulation of MMP-2 and MMP-9, suggesting impaired extracellular matrix remodeling and a reduction in regenerative potential **(C)**. DAPI staining (blue) identifies nuclei. Scale bars, 100 μm. β-actin was used as the internal control. Data are presented as mean ± SEM; $*P < 0.05$, $**P < 0.01$, $***P < 0.001$.

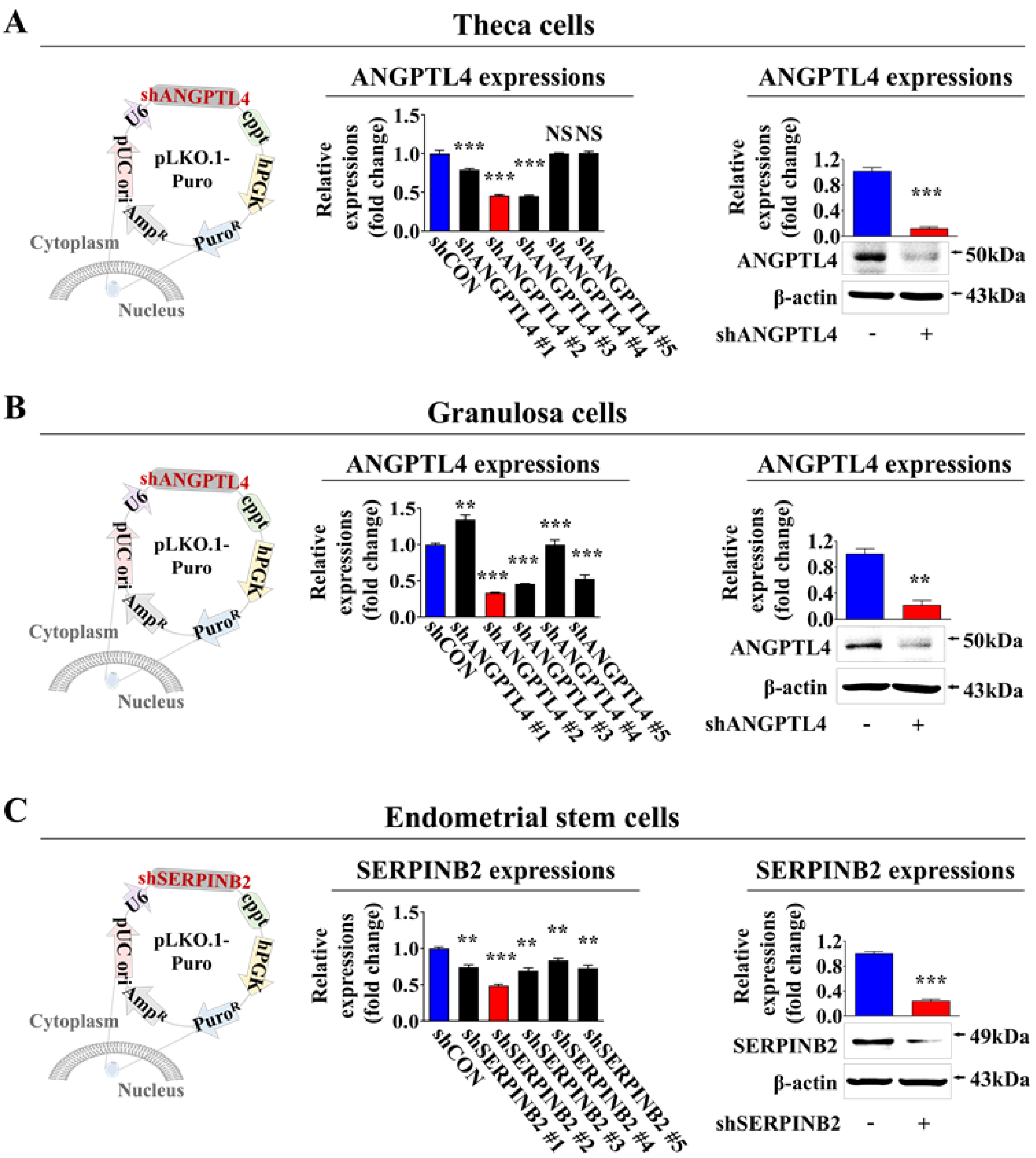

Figure S9. Targeted gene silencing of ANGPTL4 or SERPINB2 using shRNA in ovarian or endometrial cell types. Theca cells: Relative gene expression analysis by qRT-PCR demonstrated efficient knockdown of ANGPTL4 with all five independent shRNA constructs (shANGPTL4 #1–#5) when compared to the control (shCON). Among these constructs, ANGPTL4 shRNA #2, designated as ANGPTL4 shRNA in subsequent text, achieved the most pronounced silencing effect. Western blot analysis further confirmed a substantial decrease in ANGPTL4 protein expression following shRNA transfection, supporting the effectiveness of gene knockdown at both mRNA and protein levels **(A)**.

Granulosa cells: In a pattern consistent with theca cells, transfection with ANGPTL4-targeted shRNAs (shANGPTL4 #1–#5) led to significant reductions in mRNA expression, as demonstrated by qRT-PCR analysis. ANGPTL4 shRNA #2, referred to as ANGPTL4 shRNA in this context, was the most effective construct for silencing. The reduction in ANGPTL4 protein levels was also verified by immunoblot, further substantiating shRNA efficacy **(B)**. Endometrial stem cells: SERPINB2-targeted shRNA constructs (shSERPINB2 #1–#5) resulted in a marked decrease in SERPINB2 transcripts compared with shCON. Among these, SERPINB2 shRNA #2, hereafter referred to as SERPINB2 shRNA, showed superior knockdown efficiency. Western blot analysis showed decreased SERPINB2 protein expression following transfection, confirming robust gene silencing in endometrial stem cells **(C)**. β-actin was used as the internal control. Data are presented as mean ± SEM; *$P < 0.05$, **$P < 0.01$, ***$P < 0.001$.

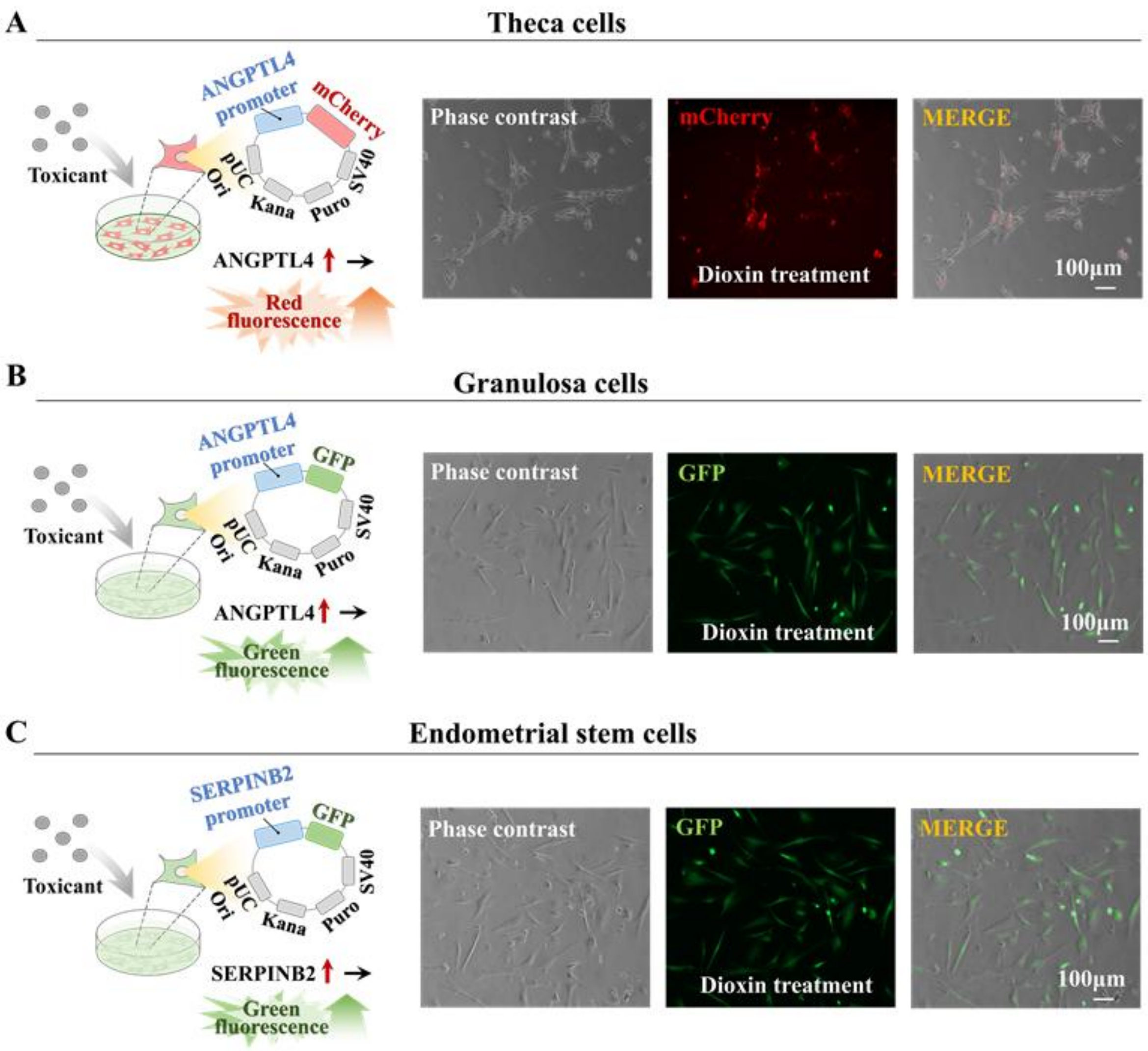

Figure S10. Development and validation of a biomarker-driven fluorescent reporter system for real-time assessment of reproductive toxicity in defined cell types. ANGPTL4 promoter-mediated mCherry reporter in theca cells. The ANGPTL4 promoter was cloned upstream of the mCherry gene and stably introduced into human theca cells. Upon treatment with a reference toxicant (dioxin), theca cells showed strong red fluorescence, demonstrating transcriptional upregulation of ANGPTL4 in response to toxicant-induced damage **(A)**. ANGPTL4 promoter-controlled GFP reporter in granulosa cells. A comparable approach was used to generate a GFP reporter under control of the ANGPTL4 promoter, which was transfected into granulosa cells. After toxicant exposure, marked green fluorescence was detected, consistent with ANGPTL4 promoter activation **(B)**. SERPINB2 promoter-driven GFP reporter in endometrial stem cells. To enable precise visualization of endometrial-specific

toxicity, the SERPINB2 promoter was fused to GFP and stably transfected into human endometrial stem cells. Toxicant challenge resulted in a substantial increase in green fluorescence in the endometrial compartment, indicating SERPINB2 promoter activation **(C)**. Overlaid fluorescence and phase-contrast images verified cell-type-specific reporter expression and structural integrity of the biosensing platform. Scale bars: 1000 μm.

Table S1. List of differentially expressed genes in response to toxic exposure in human theca cells

Gene	Gene full name	Dioxin 5ng/ml	Dioxin 7ng/ml
THBD	thrombomodulin	6.03	5.68
CYP1B1	cytochrome P450 family 1 subfamily B member 1	5.88	5.64
VIPR1	vasoactive intestinal peptide receptor 1	6.9	5.39
GRM2	glutamate metabotropic receptor 2	3.88	5.39
ANGPTL4	angiopoietin like 4	3.23	4.71
IL24	interleukin 24	6.02	4.69
SLC7A11	solute carrier family 7 member 11	3.23	4.5
PPP1R14C	protein phosphatase 1 regulatory inhibitor subunit 14C	3.03	4.4
BEX2	brain expressed X-linked 2	2.25	4.35
FAM221A	family with sequence similarity 221 member A	2.74	4.19
SERPINB2	serpin family B member 2	3.85	4.18
IL1RN	interleukin 1 receptor antagonist	5.72	4.16
ST6GALNAC2	ST6 N-acetylgalactosaminide alpha-2,6-sialyltransferase 2	2.66	4.14
TIGD3	tigger transposable element derived 3	3.46	4.12
CTSV	cathepsin V	3	4.03
AREG	amphiregulin	3.02	3.83

CCL3	C-C motif chemokine ligand 3	2.43	3.69
HUNK	hormonally up-regulated Neu-associated kinase	2.64	3.57
PLIN2	perilipin 2	2.06	3.1
REEP1	receptor accessory protein 1	N/A	11.15
LAMP3	lysosomal associated membrane protein 3	N/A	5.43
C5AR1	complement C5a receptor 1	N/A	4.29
EMILIN3	elastin microfibril interfacer 3	N/A	4.29

Table S2. List of differentially expressed genes in response to toxic exposure in human granulosa cells

Gene	Gene full name	Dioxin 5ng/ml	Dioxin 7ng/ml
RCSD1	RCSD domain containing 1	4.22	4.37
IL17RB	interleukin 17 receptor B	4.2	4.33
IL24	interleukin 24	4.14	4.44
ZNF519	zinc finger protein 519	4.07	4.48
ANGPTL4	angiopoietin like 4	2.76	3.62
LAMP3	lysosomal associated membrane protein 3	3.07	5.58
EPHA5	EPH receptor A5	5.77	5.42
THBD	thrombomodulin	5.3	5.19
ELOVL7	ELOVL fatty acid elongase 7	2.64	5
PAPPA2	pappalysin 2	3.26	4.88
RHCG	Rh family C glycoprotein	3.2	4.72
GPR1	G protein-coupled receptor 1	2.87	4.69
IL1RN	interleukin 1 receptor antagonist	3.72	4.68
TXNRD1	thioredoxin reductase 1	3.27	4.62
NLGN1	neuroligin	2.73	4.54
MCTP1	multiple C2 and transmembrane domain containing 1	5.03	4.43

SLC7A2	solute carrier family 38 member 1	3.55	4.41
CNTF	ciliary neurotrophic factor	2.24	4.26
GAS2L3	growth arrest specific 2 like 3	2.92	4.25
TMEM179	transmembrane protein 179	2.88	4.23
CREB5	cAMP responsive element binding protein 5	3.23	4.23
PLIN2	perilipin 2	2.71	4.19
SLC38A1	solute carrier family 38 member 1	2.62	4.18
INA	internexin neuronal intermediate filament protein alpha	2.39	4.17
STX11	syntaxin 11	3.01	4.11
ZC3H12C	zinc finger CCCH-type containing 12C	2.82	4.1
ZNF620	zinc finger protein 620	3.19	4.07
TP73	tumor protein p73	N/A	10.79
GRM2	glutamate metabotropic receptor 2	N/A	6.38
C5AR1	complement C5a receptor 1	N/A	5.38

Table S3. List of differentially expressed genes in response to toxic exposure in human endometrial stem cells

Gene	Gene full name	Dioxin 5ng/ml	Dioxin 7ng/ml
THBD	thrombomodulin	20.04	77.36
GPR68	G protein-coupled receptor 68	14.55	15.76
SERPINB2	serpin family B member 2	12.84	41.81
EREG	epiregulin	10.32	36.31
CLSPN	claspin	8.16	16.67
TFPI2	tissue factor pathway inhibitor 2	8.15	10.53
ISG15	ISG15 ubiquitin-like modifier	7.33	7.79
CDCP1	CUB domain containing protein 1	6.3	15.33
IFIT3	interferon induced protein with tetratricopeptide repeats 3	6.12	9.79
CCRL2	C-C motif chemokine receptor like 2	5.38	6.42
KRTAP2-3	keratin associated protein 2-3	5.28	10.66
MMP3	matrix metallopeptidase 3	5.18	28.77
STRIP2	striatin interacting protein 2	5.18	8.02
STEAP1	STEAP family member 1	5.16	6.47
HAS2	hyaluronan synthase 2	5.04	10.32
TMEM100	transmembrane protein 100	4.99	15.01

PHLDA1	pleckstrin homology like domain family A member 1	4.75	17.27
ANGPTL4	angiopoietin like 4	4.42	232.8
TK1	thymidine kinase 1	4.31	4.02
IL13RA2	interleukin 13 receptor subunit alpha 2	4.13	13.96
HSPA6	heat shock protein family A (Hsp70) member 6	N/A	2173.541
NGFR	nerve growth factor receptor	N/A	677.21
ARC	activity regulated cytoskeleton associated protein	N/A	599.355
HMOX1	heme oxygenase 1	N/A	312.936
CXCL8	C-X-C motif chemokine ligand 8	N/A	273
CXCL2	C-X-C motif chemokine ligand 2	N/A	252.891
CXCL3	C-X-C motif chemokine ligand 3	N/A	236.171
MAFB	MAF bZIP transcription factor B	N/A	161.213
FOSB	FosB proto-oncogene, AP-1 transcription factor subunit	N/A	136.365
FGF18	fibroblast growth factor 18	N/A	118.184
